# Synthesis Based on Covalent Capture and Release Enables Purification-free Fluorogenic Probe Libraries for Single-Molecule Protease Activity Profiling

**DOI:** 10.1021/acscentsci.6c00783

**Published:** 2026-06-29

**Authors:** Mayano Minoda, Tadahaya Mizuno, Takumi Iwasaka, Hiroyuki Kusuhara, Yu Kagami, Shingo Sakamoto, Norimichi Nagano, Chiaki Hori, Kazufumi Honda, Yasuteru Urano, Toru Komatsu

**Affiliations:** † Graduate School of Pharmaceutical Sciences, 13143 The University of Tokyo, 7-3-1 Hongo, Bunkyo-ku, Tokyo 113-0033, Japan; ‡ Department of Oral Pathobiological Science and Surgery, Tokyo Dental College, Chiyoda-ku, Tokyo 101-0061, Japan; § Institute for Advanced Medical Science, 26367 Nippon Medical School, 1-1-5 Sendagi, Bunkyo-ku, Tokyo 113-8602, Japan; ∥ Graduate School of Medicine, Nippon Medical School, 1-1-5 Sendagi, Bunkyo-ku, Tokyo 113-8602, Japan; ⊥ Graduate School of Medicine, The University of Tokyo, 7-3-1 Hongo, Bunkyo-ku, Tokyo 113-0033, Japan

## Abstract

Direct measurement of enzyme activities provides functional insights into complex biological systems; however, their broader application is limited by the lack of scalable strategies to generate diverse, assay-ready fluorogenic probes. In particular, conventional probe synthesis relies on chromatographic purification, preventing the rapid and parallel exploration of substrate space at the library scale. Here, we report synthesis based on covalent capture and release (SCCR), a general chemical strategy that enables purification-free generation of fluorogenic probe libraries. By embedding a covalent capture handle within a removable protecting group, SCCR establishes a standardized capture–elongation–release workflow that decouples molecular diversification from chromatographic purification while retaining the flexibility of liquid-phase synthesis. This approach enables automated preparation of high-purity probe libraries compatible with sensitive activity assays. Using this platform, we generated a library of over 100 fluorogenic probes and applied it to profile protease activities at the single-molecule level, enabling substrate discovery and the activity-based identification of disease-associated enzymatic signatures in blood samples. These results establish a scalable route to functional probe generation and expand the accessible space for single-molecule enzyme activity profiling in complex biological systems.

## Graphical abstract

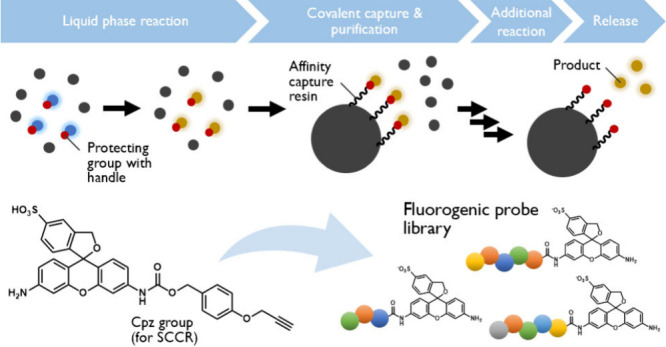

## Introduction

Living systems harbor thousands of enzymes whose functions are dynamically regulated by post-translational modifications (PTMs) and protein–protein interactions (PPIs).^1^ Because enzyme activity directly reflects functional states, its direct measurement provides a powerful means to access biologically relevant information that is often inaccessible from abundance-based analyses alone.^2–4^ A central challenge in this area is the preparation of fluorogenic probes that meet the stringent requirements for sensitive activity measurements. In particular, probe performance in high-resolution assays, such as single-molecule enzyme activity profiling (SEAP),^5,6^ is highly dependent on chemical purity, as even trace amounts of fluorescent impurities can lead to elevated background signals and obscure low-turnover enzymatic events. Conventional synthetic workflows typically rely on chromatographic purification to achieve assay-ready quality, but this requirement fundamentally limits throughput and scalability. As a result, the rapid and parallel generation of diverse probe libraries, which is essential for exploring the broad substrate space of enzymes, remains difficult.

To address this challenge, we developed synthesis based on covalent capture and release (SCCR), a general and automation-compatible chemical strategy (Figure 1a). In this approach, a covalent capture handle is embedded within a removable protecting group, enabling intermediates to be selectively immobilized, transformed, and subsequently released through a standardized capture–elongation–release workflow. This design decouples molecular diversification from chromatographic purification while retaining the versatility of liquid-phase reactions.

**Figure 1:**
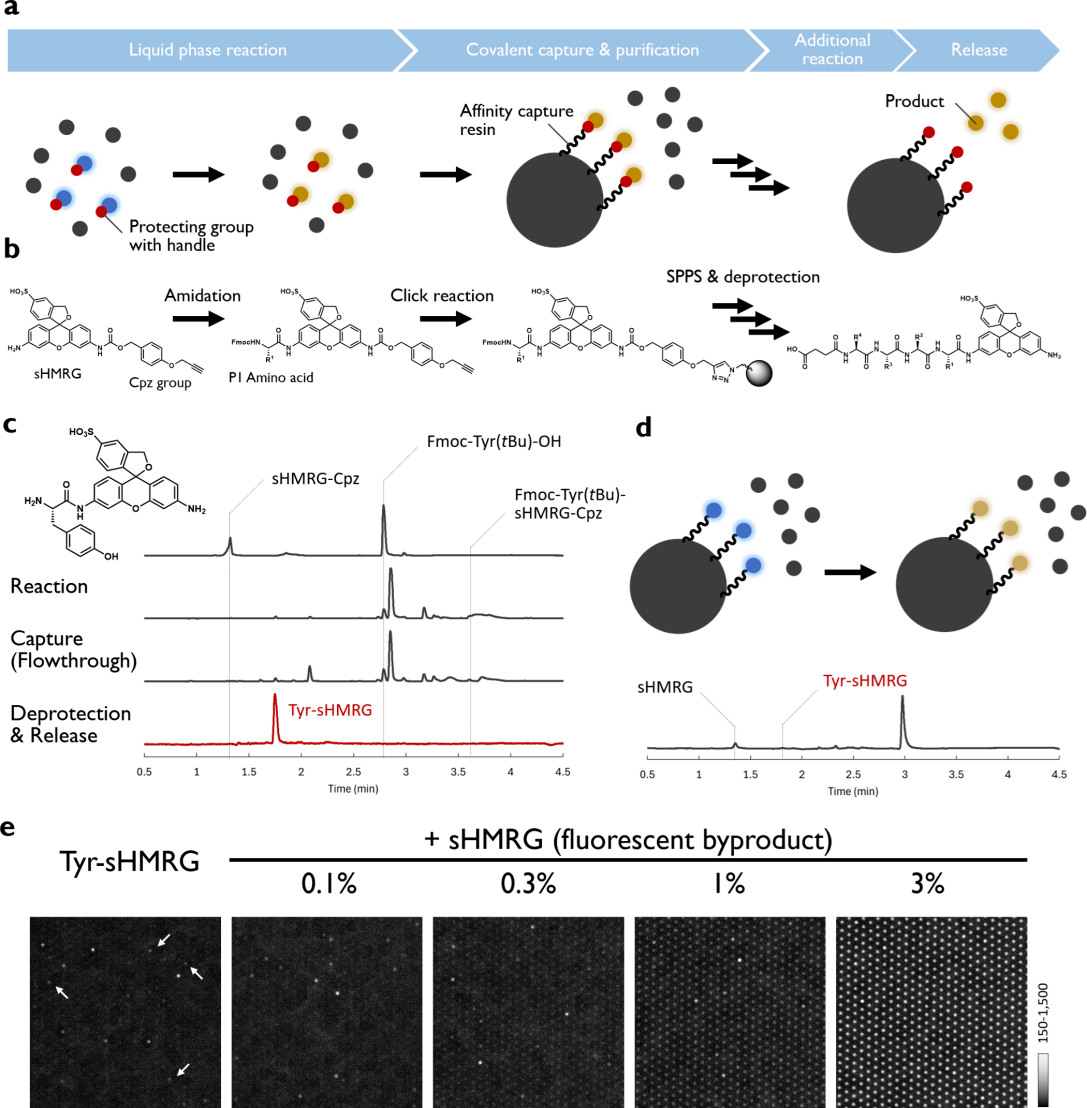
Concept and workflow of synthesis based on covalent capture and release (SCCR). (a) Conceptual workflow of SCCR by covalently capturable protecting groups. (b) Molecular structure of Cpz-modified sHMRG designed for SCCR-based preparation of fluorogenic probes for proteases. (c) LC chromatograms (254 nm absorbance) monitoring the preparation of Tyr-sHMRG using SCCR. The reaction was performed by mixing sHMRG-Cpz (1 μmol), Fmoc-Tyr­(*t*Bu)-OH (10 μmol), DMT-MM (10 μmol), and DIEA (15 μmol) in NMP (15 μL) and DCM (500 μL) at 65 °C for 2 h. After the capture in click reaction conditions with azide resin and deprotection of Fmoc group, the probe was cleaved using 90% TFA-10% H_2_O. (d) LC chromatograms (254 nm absorbance) monitoring the preparation of Tyr-sHMRG using sHMRG and CTC resin. sHMRG (20 μmol) in DIEA (100 μL) and DMSO (200 μL) was captured by CTC resin (60 mg) by reacting at 25 °C for 2 h, and after washout, it was reacted with Fmoc-Tyr­(*t*Bu)-OH (0.2 M), DMT-MM (0.2 M) in DIEA-NMP (1:2, 500 μL) for 6 h. After deprotection of Fmoc group on solid support, the probe was cleaved using 90% TFA-10% H_2_O. (e) Fluorescence images of microdevice after loading Tyr-sHMRG (30 μM) containing a varied ratio of sHMRG (0–3%, 0–900 nM) and plasma samples of healthy human subjects (1/5000 dilution) in HEPES-Na buffer (100 mM, pH 7.4) containing CaCl_2_ (1 mM), MgCl_2_ (1 mM), DTT (100 μM), and Triton X-100 (150 μM) and incubating at 25 °C for 2 h. Fluorescence spots indicated by white arrows indicate the enzymes with a lower turnover number.

### Establishment of SCCR Scheme for Preparation of Fluorogenic Probes without Chromatographic Purification

In this study, we focused on proteases as representative targets, as their activities are involved in a wide range of biological processes and diseases,^7,8^ and peptide-based fluorogenic probes provide a direct readout of enzymatic activity. A central synthetic challenge in constructing such probes lies in the attachment of the substrate peptide scaffold to the aniline moiety of the fluorophore (Figure 1b).^6^ Because unreacted fluorophores remain intrinsically fluorescent, even trace amounts of unconverted starting materials lead to elevated background signals that interfere with sensitive activity measurements, particularly at the single-molecule level. However, this transformation often proceeds with an incomplete conversion, especially under heterogeneous conditions.

Existing strategies, including solid-phase synthesis^9,10^ and partial solution-phase approaches,^11^ have been explored to address this issue, but they generally fail to simultaneously achieve the high chemical purity required for sensitive assays and the scalability needed for library generation (Figure S1). We previously developed synthesis based on affinity separation (SAS), in which liquid-phase intermediates are equipped with an affinity handle for purification.^12,13^ While effective, this approach requires a permanent handle, limiting its applicability to structurally diverse fluorophore scaffolds. To overcome these limitations, we envisioned a strategy in which the capture handle is embedded within a removable protecting group, enabling the covalent capture-based purification and traceless release.^12^ To implement this concept, we designed the *p*-propargyloxybenzyl (Pzl) group based on the acid-labile *p*-methoxybenzyl (PMB) scaffold (Figure 1b, Scheme S1). The propargyl moiety enables rapid and selective covalent capture via click chemistry with azide-functionalized solid supports. As an amine protecting group, Pzl forms a carbamate (Cpz), analogous to Cbz, and can be removed under acidic conditions to release the final product without residual modification. This design enables a capture–elongation–release workflow in which intermediates are selectively immobilized, transformed, and ultimately released in high purity.

To construct fluorogenic probe libraries for single-molecule enzyme activity analysis, we employed the sHMRG fluorophore^6,14^ and prepared sHMRG-Cpz as a common building block (Figure 1b, Schemes S1 and S3). The synthetic sequence was optimized as follows. The initial amidation of the P1 amino acid in the liquid phase achieved near-quantitative conversion under conditions of excess reagents (5 equiv), high concentration (1 M), and elevated temperature (55–65 °C), conditions that are difficult to realize in conventional solid-phase synthesis.^12^ The resulting intermediate was then efficiently captured using PEGylated azide-functionalized TentaGel-NH_2_ beads (Scheme S2), achieving quantitative immobilization within 15 min (Figure S2a,b). This process was significantly faster and more efficient than conventional loading onto 2-chlorotrityl chloride (CTC) resin, which required prolonged reaction times and excess reagents. Importantly, the azide-functionalized resin selectively captured the Cpz-bearing scaffold directly from the crude reaction mixture, effectively removing excess reagents and byproducts (Figure 1b). Following capture, the Cpz group remained stable during Fmoc deprotection and subsequent peptide elongation. Finally, cleavage of the protecting group under standard acidic conditions (90% TFA in H_2_O) afforded the desired product with near-quantitative recovery within 90 min (Figure S2c).

As a proof of concept, we examined the preparation of Tyr-sHMRG as a representative fluorogenic probe. The SCCR workflow proceeded as designed through reaction, capture, and release steps (Figure 1c), affording the desired product with high purity (>99%, monitored at 254 nm) without chromatographic purification. In contrast, attempts to prepare the same probe using conventional CTC-based solid-phase synthesis failed, primarily due to incomplete conversion and nonselective adsorption of reagents on the solid support, preventing the generation of assay-ready material (Figure 1d). The SCCR-synthesized probe enabled the detection of the single-molecule activities of various aminopeptidases in blood samples. Notably, even minor levels of fluorescent byproducts (greater than 1%) produced substantial background signals in microchambers, obscuring low-turnover enzymatic events (Figure 1e). These results highlight the stringent purity requirements for fluorogenic probes in single-molecule assays and underscore the importance of purification-free strategies that can reliably deliver assay-ready materials.

Encouraged by these results, we next explored the scalability of the SCCR workflow. Owing to its simplicity and modularity, the entire process was readily automated using a peptide synthesizer, enabling the preparation of a library of 103 probes targeting diverse endo- and exopeptidases (Figure 2a, Table S1, Scheme S3, Figures S3 and S4). Across the library, fluorescent byproducts were consistently suppressed to below 1% after release from the azide-functionalized resin, providing assay-ready probe quality without chromatographic purification.

**Figure 2:**
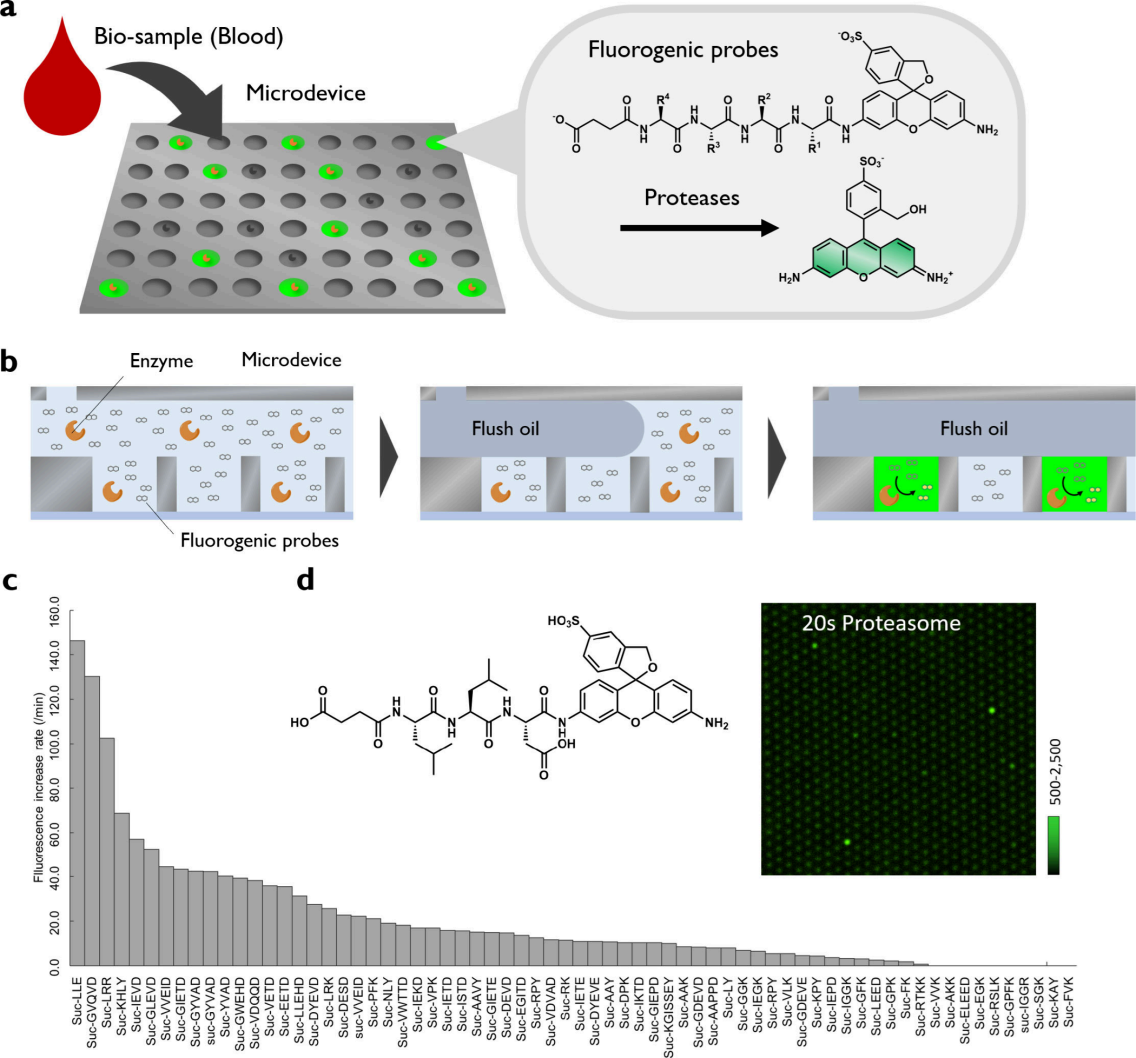
Establishment of activity-based screening to characterize single-molecule enzyme activities of recombinant enzymes and biomarker enzymes in blood samples. (a) Assay principle of single-molecule enzyme activity profiling (SEAP) using fluorogenic probes for proteases and microdevice. (b) Workflow of the SEAP assay. (c) Substrate profiling of 20S proteasome. Fluorogenic probes (10 μM) were mixed with proteasome 20S (activated by Triton X-100, 1 μg/mL) in HEPES-Na buffer (100 mM, pH 7.4) containing CHAPS (0.1%) and ATP (3 mM) and incubated for 60 min. The slope of the fluorescence increase was calculated. (d) Single-molecule enzyme activity assay of proteasome 20S. Suc-LLE-sHMRG (30 μM) was mixed with proteasome 20S (10 ng/mL) in HEPES-Na buffer (100 mM, pH 7.4) containing CaCl_2_ (1 mM), MgCl_2_ (1 mM), DTT (100 μM), and Triton X-100 (150 μM) and incubated at 25 °C for 18 h. Fluorescence images were acquired using an epifluorescence microscope.

In a small subset of cases (8/103), higher levels of impurities (>10%, monitored at 500 nm) were observed. These impurities predominantly originated from peptide elongation steps, reflecting known limitations of peptide synthesis such as methionine oxidation and incomplete side-chain deprotection rather than the SCCR process itself. When necessary, these cases were readily addressed by rapid preparative MPLC, ensuring the overall quality and usability of the probe library (Table S1). For probes containing amino acids with acid-labile side-chain protecting groups that generate nonvolatile fragments (e.g., Pbf and Trt), residual byproducts remained after cleavage (Figure S5a,b); however, we used them for screening purposes without additional purification since the residual products do not generate fluorescent signals. The yields of representative probes ranged from 10% to 70% (based on the starting material sHMRG-Cpz; Figure S5c). This recovery likely reflects the efficient covalent capture and release processes of the present platform (Figure S2b,c).

### Substrate Profiling of Recombinant Proteasome 20S Using Single-Molecule Enzyme Activity Assays

Proteases constitute a large and diverse class of enzymes with distinct substrate preferences, particularly at the P1 position (Table S2). Despite their broad biological and clinical importance, many proteases still lack suitable fluorogenic probes for activity analysis. This limitation is especially pronounced for highly sensitive single-molecule measurements (Figure 2a and b), where probe performance critically depends on chemical purity and signal-to-noise characteristics are in severe lack. Among such targets, the proteasome represents a prominent and biologically important example. The proteasome is a large multisubunit protease complex responsible for regulated protein degradation and plays essential roles in diverse cellular processes, including protein quality control and antigen presentation.^15–17^ The 20S core particle exhibits multiple catalytic activities, commonly classified as caspase-like, trypsin-like, and chymotrypsin-like, which are further modulated by various PTMs.^15^ Although previous studies have identified peptide sequences recognized by the proteasome,^16^ the fluorophore scaffold can significantly influence enzyme recognition, and thus suitable fluorogenic probes cannot be reliably predicted from peptide sequences alone. Therefore, we performed substrate profiling using our probe library and recombinant human proteasome 20S, identifying multiple substrates that are efficiently metabolized by the proteasome (Figure 2c). The hit probes included the probes with known proteasome substrate sequences, such as Suc-Leu-Leu-Glu (LLE) and Suc-Leu-Arg-Arg (LRR), which preferentially report caspase-like and trypsin-like activities, respectively.^16^ In addition, the profiling revealed previously unrecognized sequences, including Suc-Lys-His-Leu-Tyr (KHLY), which exhibited higher chymotrypsin-like activity than the commonly used Suc-Ala-Ala-Val-Tyr (AAVY) motif, and Suc-Gly-Val-Gln-Val-Asp (GVQVD), a sequence originally developed for caspase-3 and -6, that also reported caspase-like proteasome activity. The most effective substrate among them was Suc-LLE, which successfully reported proteasome activity on the SEAP platform (Figure 2d). These results demonstrate that SCCR-enabled probe libraries can efficiently identify functional substrates, thereby enabling the development of single-molecule assays for complex proteases. This validation in a defined recombinant system motivated us to apply the same probe library to complex biological samples.

### Activity-Based Screening to Identify Specific Biomarker Activities of Liver Damage

Having established that the SCCR-enabled probe library enables rapid identification of functional substrates and single-molecule measurements in a recombinant system, we next asked whether the same library could extract biologically meaningful enzyme activity alterations from complex biological samples. Activity-based screening using enzymomics approaches has proven useful for discovering functional biomarkers by comparing enzyme activity signatures across biological states.^12,14,18^ As a proof of concept, we searched for blood-based activity biomarkers of liver damage.

Two mouse models of liver damage with distinct molecular mechanisms were examined. The thioacetamide (TAA) model reflects hepatic cell damage, whereas the 4,4’-methylene dianiline (MDA) model reflects cholestasis.^18,19^ The current biomarker, aspartate/alanine aminotransferase (AST/ALT), cannot reliably discriminate between these two liver disease models,^19^ but we hypothesized that monitoring unique changes in enzyme activity patterns could distinguish the different pathologies.

Screening was performed using 44 representative probes selected to broadly cover major protease classes, and some of the probes showed notable differences in enzyme activities between control (vehicle-treated), TAA-treated, and MDA-treated mice (Figure 3a, Figure S6). In TAA-treated mice, enzymes responding to Suc-Glu-Leu-Glu-Val-Asp (GLEVD)-sHMRG were upregulated (Figure 3b and c). The sequence is a substrate of caspases, and other probes targeting caspases, such as Suc-WEHD, Suc-GYVAD, Suc-IETD, and Suc-EETD, also showed increased spot numbers in TAA-treated mice (Figure 3a). TAA damages hepatic cells,^19^ and these results thus indicated the occurrence of apoptosis in liver tissues, resulting in leakage of caspases into the circulation. In MDA-treated mice, enzymes responding to Suc-Lys-His-Leu-Tyr (KHLY)-sHMRG (targeting kallikreins) appeared to be upregulated (Figure 3a). There were multiple spots with distinctive activities, indicating the presence of multiple enzyme species. Because it was difficult to distinguish the resembling activity species using a single-color assay, we sought to construct multicolored SEAP assays^5,6^ by introducing a second fluorogenic probe with a distinct color using the SCCR platform.

**Figure 3:**
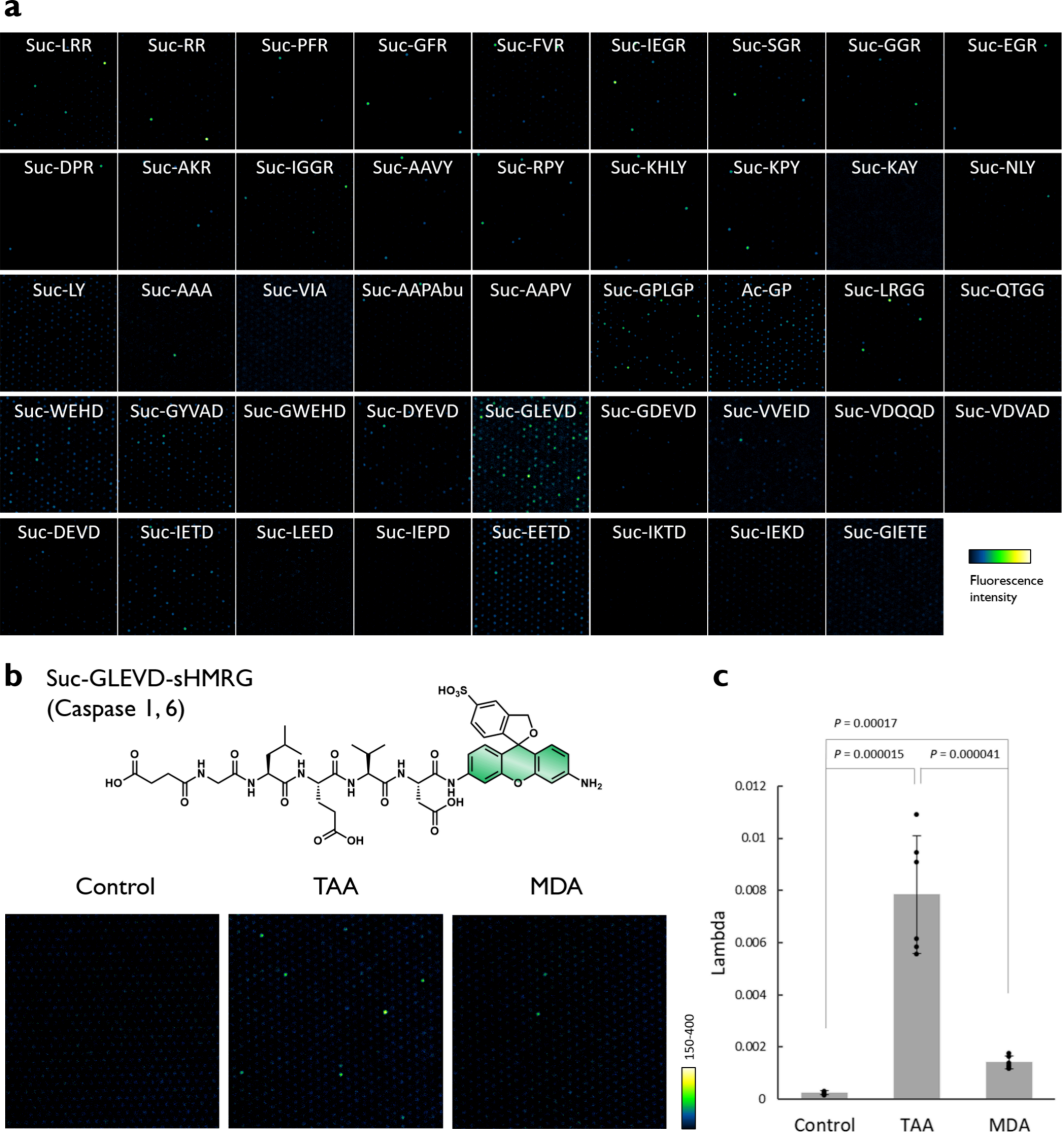
Single-molecule protease activity-based screening. (a) Result of activity-based screening of potential biomarkers of liver damage. The assay was performed by mixing probes (30 μM) with plasma samples from TAA-treated mice (1/100, 1/1,000 for pro-endopeptidases) in HEPES-Na buffer (100 mM, pH 7.4) containing CaCl_2_ (1 mM), MgCl_2_ (1 mM), DTT (1 mM), and Triton X-100 (250 μM) and incubating at 25 °C for 18 h. Full data set acquired as a comparison with blood samples from control mice and MDA-treated mice is shown in Figure S6. (b) Fluorescence images of the microdevice after loading Suc-GLEVD-sHMRG (30 μM) and plasma samples (1/3,000) in HEPES-Na buffer (100 mM, pH 7.4) containing CaCl_2_ (1 mM), MgCl_2_ (1 mM), DTT (1 mM), and Triton X-100 (250 μM) and incubating for 18 h at 25 °C. (c) Quantification of bright spots in (b); *n* = 4 for control and *n* = 6 for TAA- or MDA-treated mice. Error bars represent S.D. *P* values were calculated using Student’s *t* test.

### Multicolored Extension of the Library Approach

To enable multicolor single-molecule activity profiling, we extended the SCCR strategy to red-emitting fluorophore scaffolds. We developed disulfonated Si-rhodamine (dsSiR) as a red fluorescent fluorophore with enhanced hydrophilicity compared to previously developed sSiR^6^ (Scheme S4). One synthetic hurdle was that, unlike sHMRG, the dsSiR scaffold cannot undergo spirolactam formation, rendering carbamate-based protection (e.g., Cpz) unsuitable for initial amidation due to reduced reactivity of the second amino group in the open-ring form (Figure S7). To address this, we employed the leuco form of dsSiR and designed an alkyl-type SCCR-compatible protecting group, ethynyl-Trt (Ert). Using leuco dsSiR-Ert as a common building block, probe synthesis proceeded in a manner largely analogous to that of sHMRG-Cpz. Although this scheme required one additional step, oxidation of the leuco dsSiR after peptide elongation, this reaction could also be performed on the solid phase, allowing the overall workflow to remain largely unchanged and compatible with automated procedures (Figure 4a).

**Figure 4:**
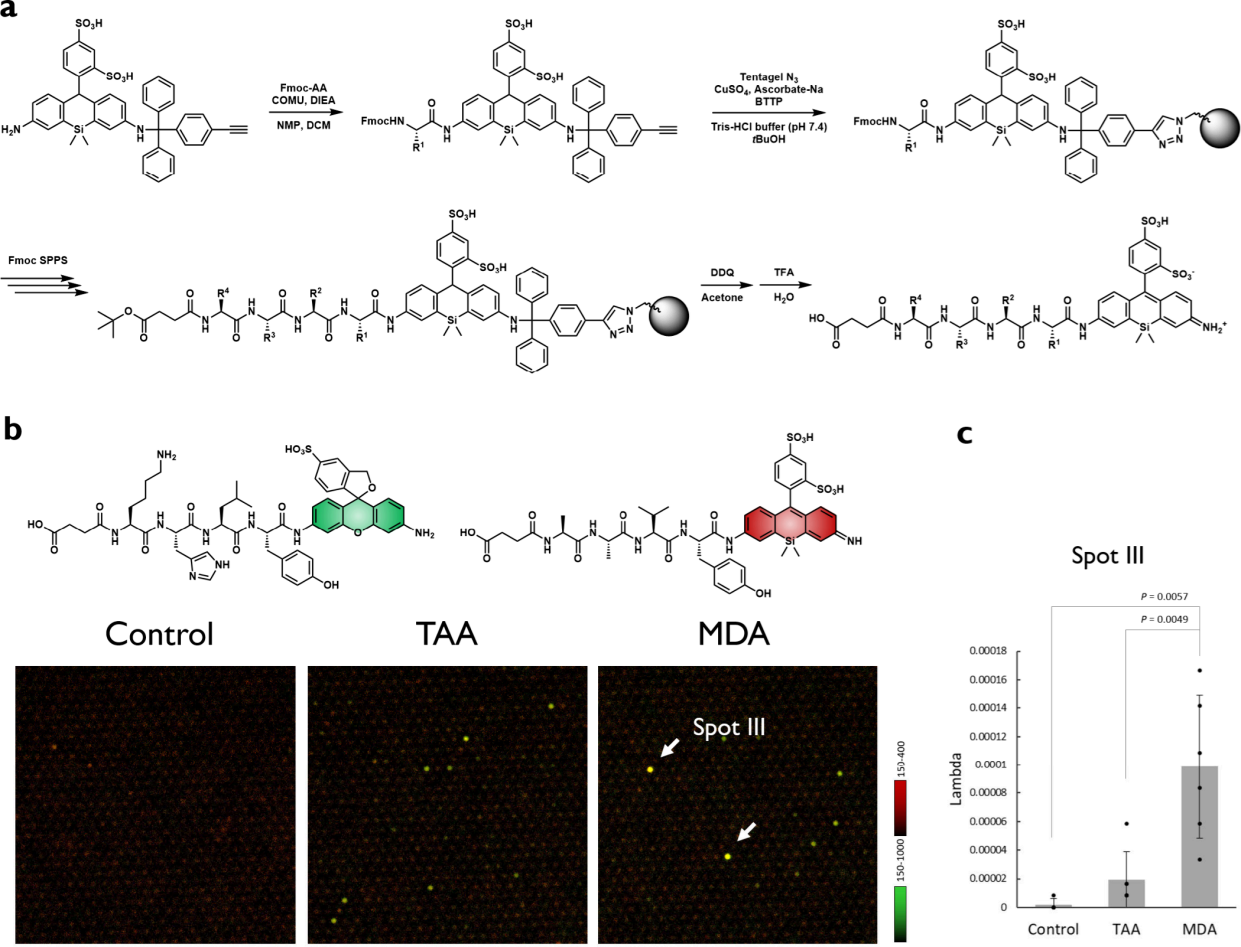
Construction of multicolored SEAP assay as an extension of SCCR workflow. (a) Scheme of preparing dsSiR-based fluorogenic probe using SCCR workflow. (b) Fluorescence images of the microdevice after loading Suc-KHLY-sHMRG (30 μM), Suc-AAVY-dsSiR (30 μM) and plasma samples (1/100) in HEPES-Na buffer (100 mM, pH 7.4) containing CaCl_2_ (1 mM), MgCl_2_ (1 mM), DTT (1 mM), and Triton X-100 (250 μM) and incubating for 18 h at 25 °C. The scattered plot analysis of the activity spots is shown in Figure S7a. White arrows indicate the enzymes having strong activities against both substrates (spot III). (c) Quantification of enzymes in spot III. *n* = 4 for control and *n* = 6 for TAA- or MDA-treated mice. Error bars represent S. D. *P* values were calculated using Student’s *t* test. Full data set is shown in Figure S8b.

As a general probe targeting chymotrypsin-like activities, we prepared the fluorogenic probe with the Suc-AAVY sequence. The probe pair consisting of Suc-KHLY-sHMRG (green) and Suc-AAVY-dsSiR (red) revealed three distinct activity clusters in blood samples, and significant increases of enzyme spots exhibiting high activity toward both probes were observed in MDA-treated mice (Figures 4b,c and S8). Treatment with MDA triggers the activation of various immune cells accompanying cholestasis;^19^ thus, we hypothesize that it may reflect the upregulation of specific kallikrein-related activities in response to the activation of these cells. Although a complete understanding of the molecular background will require further investigation, these results demonstrate that SCCR enables the systematic construction of multicolor probe sets, thereby expanding the dimensionality of single-molecule enzymatic profiling and enhancing the resolution of activity-based biomarker discovery.

## Discussion

In this study, we establish synthesis based on covalent capture and release (SCCR) as a general chemical strategy that enables purification-free generation of fluorogenic probe libraries readily applicable to highly sensitive enzyme activity assays, including single-molecule enzyme activity profiling (SEAP). By embedding a covalent affinity handle within a removable protecting group, SCCR overcomes a key limitation of conventional workflows in which chromatographic purification is intrinsically coupled to molecular diversification.

A key enabling feature of SCCR lies in the design of protecting groups that integrate a covalent capture functionality into conventional protection chemistry. In this study, we developed three SCCR-compatible protecting groups, Cpz, Pzl, and Ert, which demonstrate how covalent capture and traceless release can be incorporated into removable protecting groups while maintaining compatibility with established synthetic transformations (Figure S9). Although the present work focuses on acid-labile protecting groups because of their compatibility with Fmoc-based peptide synthesis, the underlying design principle should be applicable to a broader range of protecting group classes. This design uniquely combines the high reactivity and flexibility of liquid-phase synthesis with the operational simplicity of solid-phase purification, enabling multistep, purification-free workflows. Beyond fluorogenic protease probes, the SCCR concept should be adaptable to other enzyme classes, enzyme-activatable chemical tools, and potentially a wider range of functional small molecules. By enabling the scalable and automated generation of high-purity probe libraries, SCCR lowers the technical barriers associated with complex chemical synthesis and broadens access to custom chemical tools across life sciences and biomedical research.

The library of 103 probes prepared in this study provides a versatile resource for developing SEAP assays and identifying activity-based biomarkers. Although liver damage models were used here as a proof-of-concept, the screening strategy is readily applicable to a wide range of biological samples, including human clinical specimens.^5,12,14^

Beyond the identification of specific enzymes, a key advantage of activity-based profiling at the single-molecule level is that changes in activity patterns themselves can serve as functional biomarkers.^6^ While assigning these activities to specific enzyme species remains an important challenge, this framework provides a direct route to linking enzymatic functions with disease states.

Taken together, SCCR establishes a scalable and general strategy for generating high-purity chemical libraries, thereby expanding the accessible space of functional probe discovery and enabling new modes of single-molecule enzymatic analysis in complex biological systems.

## Conclusion

We have established a synthesis based on covalent capture and release (SCCR) as a general chemical strategy in which a covalent capture handle is embedded within a removable protecting group, enabling purification-free generation of high-purity fluorogenic probe libraries. This design overcomes a key limitation of conventional workflows by decoupling molecular diversification from chromatographic purification, thereby enabling scalable and automated probe synthesis. Using this platform, we enabled the single-molecule activity profiling of proteases and identified candidate activity-based biomarkers in disease models. More broadly, SCCR provides a general framework for scalable chemical synthesis, expanding access to high-quality functional probes and enabling new modes of enzymatic analysis in complex biological systems.

## Methods

### Plasma Samples from Healthy Human Subjects

Plasma samples were collected from the Kagoshima Prefectural Comprehensive Health Center with the Program for Promotion of Fundamental Studies in Health Sciences conducted by the National Institute of Biomedical Innovation of Japan, Health and Labour Sciences Research Grants from the Ministry of Health, Labour and Welfare of Japan, and P-CREATE of the Japan Agency for Medical Research and Development (AMED).^20–22^ Ethical approval for this study was obtained from the central ethics committee of Nippon Medical School (M-2021-002), the ethical committee of Nippon Medical School (A-2020-032 and A-2020-044), and the ethical committee of The University of Tokyo (2-16 and 3-9).

### Plasma Samples from Mice

Ethical approval for the study using animals was obtained from the Animal Care and Use Committee of The University of Tokyo (P4-21, P31-9). Six-week-old male C57BL/6JJcl mice were purchased from CLEA Japan (Tokyo, Japan) and acclimatized for 5 days. The mice were exposed to thioacetamide (TAA, T0817, Tokyo Chemical Industry Co., Japan, 350 mg/L) or 4,4’-methylene dianiline (MDA, M0220, Tokyo Chemical Industry Co., Japan, 750 mg/L) dissolved in drinking water to induce liver damage, whereas control mice received tap water. After 4 days of treatment, mice were euthanized and blood was collected from the inferior vena cava into 1.5 mL tubes containing 1.5 μL heparin (Yoshindo Inc., Japan). The collected blood sample was centrifuged (1,700 g, 4 °C for 15 min) for plasma separation. Characterization of the blood samples and the detailed states of mice are shown in the cited refs. 18,19

### Instruments

NMR spectra were recorded on a JEOL JNM-LA400 instrument at 400 MHz for ^1^H NMR and 100 MHz for ^13^C NMR. Mass spectra (MS) were measured with a JEOL JMS-T100LC AccuToF (ESI). LC-MS analyses were performed on a Waters Acquity UPLC (H Class)/QDa quadrupole MS analyzer or an Acquity UPLC (H Class)/Xevo TQD quadrupole MS/MS analyzer equipped with an Acquity UPLC BEH C18 column (Waters). Column chromatography using silica gel was performed on an MPLC system (Yamazen Smart Flash EPCLC AI-5805 (Tokyo, Japan)). Reversed-phase MPLC purification was performed on an Isolera One (Biotage) equipped with a SNAP Ultra C18 30 g column (Biotage).

### LC-MS Analysis

LC-MS analyses were performed using a binary gradient of A:B = 99:1 isocratic for 0.3 min, followed by 99:1 to 0:100 (linear) over 3.2 min at a flow rate of 0.8 mL/min; solvent A consisted of H_2_O + 0.1% trifluoroacetic acid (TFA), and solvent B consisted of 80% acetonitrile/20% H_2_O + 0.1% TFA. The purity of the probes was determined by integrating the peak areas of chromatograms monitored at 500 nm; the percentage of the desired product peak area relative to the total integrated area (the desired peak plus notable impurities) was calculated, excluding the solvent front and injection artifacts.

### Preparation of Proteasome 20S

Recombinant proteasome 20S (R&D Biosystems, E-360, Lot #DBGH0724071) was activated by incubating the protein (10 μg/mL) in HEPES-Na buffer (100 mM, pH 7.4) containing Triton X-100 (0.03%) at 25 °C for 30 min. The enzyme was diluted to the indicated concentrations for assays.

### Digital Enzyme Assay in Microdevice

Digital enzyme assays were performed using commercially available microdevices^6,23^ (Simoa disk; Quanterix). A 40 μL of mixture of enzyme and reagents in buffer was loaded into the microdevice by manual pipetting. Then, 80 μL of FC-70 (Sigma-Aldrich) was introduced into the device to flush out excess reaction mixture. The enzymatic activity in the chambers was measured using an epifluorescence microscope (Ti2, Nikon) equipped with a 20× dry objective lens (Plan Apo 20×), an sCMOS camera (ORCA-Fusion C14440, Hamamatsu Photonics), a white LED illumination unit (X-Cite Xylis, Opto Science), and a motorized stage. The sHMRG-based assay was performed using a solution containing dsSiR (10 μM) as the internal standard, and the focus was adjusted using its fluorescence. Images were acquired in tile scan mode with perfect focus. The excitation and emission filters used were FITC (mirror = 510 nm, Ex. = 460–500 nm, Em. = 510–560 nm) and mCherry (mirror = 600 nm, Ex. = 550–590 nm, Em. = 608–683 nm), respectively.

### Image Processing

Images were processed using the GA3 module of NIS Elements software (Nikon). First, all fluorescence images were background-corrected using a rolling ball correction (3 μm). Then, ROIs were chosen by bright spot detection using mCherry filter settings (diameter = 3 μm, contrast = 500), and irregular fluorescent spots derived from fluorescent debris or air bubbles were omitted by dilating the ROI and removing the overlapping ROIs. The fluorescence signals were calculated as the mean of the signals from the center of each ROI. Data were processed using Excel, or Kaleidagraph software to construct histograms and scatter plots.

## Supplementary Material



## Notes

Supplementary experimental results and supplementary methods for synthesis and characterization of compounds (PDF)

## #

(M.M.) Cosomil, Inc., 7-3-1 Hongo, Bunkyo-ku, Tokyo 113-8485, Japan

## ∇

(Y.K.) Cosomil, Inc., 7-3-1 Hongo, Bunkyo-ku, Tokyo 113-8485, Japan

## □

(S.S.) Cosomil, Inc., 7-3-1 Hongo, Bunkyo-ku, Tokyo 113-8485, Japan
