# Supplementary material for: Synthesis Based on Covalent Capture and Release Enables Purification-free Fluorogenic Probe Libraries for Single-Molecule Protease Activity Profiling
Source: ACS Cent Sci. 2026 Jun 29;12(7):1048–56. doi: 10.1021/acscentsci.6c00783 (PMC13397433; doi:10.1021/acscentsci.6c00783)
Supplement: Supplementary file 1 [file oc6c00783_si_001.pdf]

Supporting Information

Synthesis based on covalent capture and release enables purification-free fluorogenic probe libraries for single-molecule protease activity profiling

Mayano Minoda, Tadahaya Mizuno, Takumi Iwasaka, Hiroyuki Kusuhara, Yu Kagami, Shingo Sakamoto, Norimichi Nagano, Chiaki Hori, Kazufumi Honda, Yasuteru Urano, and Toru Komatsu

Contents

Supplementary table S1-S2

Supplementary figure S1-S9

Supplementary methods for synthesis and characterization of compounds

Supplementary references

Table S1 List of sHMRG-based fluorogenic probes used in screening

#	Sequence	Potential target	Preparation (※1)	References (※2)
1	Suc-LRR	Proteasome trypsin-like	SCCR	*ChemMedChem* **2010**, *5*, 1236-1241
2	Suc-RR	Cathepsin B	SCCR	*Biochemistry* **2022**, *61*, 1904-1914
3	Suc-PFR	Kallikrein	RP-MPLC	*Rinsho Kagaku* **1981**, *19*, 140-148
4	Suc-GFR	Plasma kallikreins	SCCR	*J. Biochem.* **1977**, *82*, 1495-1498
5	Suc-GPFR	Pancreatic kallikreins, Plasmin	SCCR	*J. Biochem.* **1977**, *82*, 1495-1498
6	Suc-FVR	Thrombin	SCCR	*Chem. Bioll. Drug Des.* **2006**, *68*, 11-19
7	Suc-IEGR	Factor Xa	SCCR	*J. Biochem.* **1977**, *82*, 1495-1498
8	Suc-SGR	Factor Xa	SCCR	*J. Biochem.* **1977**, *82*, 1495-1498
9	Suc-GGR	Urokinase	SCCR	*J. Biochem.* **1977**, *82*, 1495-1498
10	Suc-EGR	Urokinase	SCCR	*J. Biochem.* **1977**, *82*, 1495-1498
11	Suc-DPR	Tryptase	SCCR	*Bioorg. Med. Chem.* **2002**, *10*, 3637-3647
12	Suc-AKR	Neurohypophysial granule endopeptidase	SCCR	*Biochem. Biophys. Res. Commun.* **1992**, *183*, 128-137
13	Suc-IGGR		SCCR	
14	Suc-AKK		SCCR	
15	Suc-DPK		SCCR	
16	Suc-FVK		SCCR	
17	Suc-GPK	Plasmin	SCCR	*J. Biochem.* **1980**, *88*, 183-190
18	Suc-RSLK	Site 1 Protease	RP-MPLC	*J. Biol. Chem.* **1999**, *274*, 22805-22812
19	Suc-VLK	Calpain, Regmain	SCCR	*J. Biol. Chem.* **1984**, *259*, 12489-12494
20	Suc-LRK		SCCR	
21	Suc-PFK		SCCR	
22	Suc-RTKK		SCCR	
23	Suc-IGGK		SCCR	
24	Suc-GGK		SCCR	
25	Suc-EGK		SCCR	
26	Suc-VPK		SCCR	
27	Suc-IEGK		SCCR	
28	Suc-SGK		SCCR	
29	Suc-GFK		SCCR	
30	Suc-GPFK		SCCR	
31	Suc-RK		SCCR	
32	Suc-FK		SCCR	
33	Suc-VVK		SCCR	
34	Suc-AAK		SCCR	
35	Suc-AAVY	Proteasome chymotrypsin-like	SCCR	*ChemMedChem* **2010**, *5*, 1236-1241
36	Suc-KGISSEY	Kallikrein 2	SCCR	*Biochem. Biophys. Res. Commun.* **1997**, *238*, 549-555
37	Suc-RPY	Kallikrein 3 (PSA)	SCCR	*J. Biol. Chem.* **1997**, *272*, 21582-21588
38	Suc-KHLY	Kallikrein 7	SCCR	*J. Invest. Dermatol.* **2017**, *137*, 430-439
39	Suc-LLVY	Chymotrypsin, Ingensin, Proteasome, Calpain	SCCR	*ChemMedChem* **2010**, *5*, 1236-1241
40	Suc-KPY		SCCR	
41	Suc-KAY		SCCR	
42	Suc-NLY		SCCR	
43	Suc-LY		SCCR	
44	Suc-AAY		RP-MPLC	
45	Suc-WEHD	Caspase-1	SCCR	*J. Biol. Chem.* **1997**, *272*, 9677-9682
46	Suc-GYVAD	Caspase-1	SCCR	*J. Biol. Chem.* **1997**, *272*, 9677-9682
47	Suc-GWEHD	Caspase-1	SCCR	*J. Biol. Chem.* **1997**, *272*, 9677-9682
48	Suc-YVAD	Caspase-1	SCCR	*Chem. Pharm. Bull.* **1995**, *43*, 1336-1339
49	Suc-DYEVD	Caspase-1, Caspase-3, Caspase- 7	SCCR	*J. Biol. Chem.* **1997**, *272*, 9677-9682
50	Suc-GLEVD	Caspase-1, Caspase- 6	SCCR	*J. Biol. Chem.* **1997**, *272*, 9677-9682
51	Suc-GDEVD	Caspase-1, Caspase- 3, Caspase-7	SCCR	*J. Biol. Chem.* **1997**, *272*, 9677-9682
52	Suc-VVEID	Caspase-1, Caspase-3, Caspase-6, Caspase-7	SCCR	*J. Biol. Chem.* **1997**, *272*, 9677-9682
53	Suc-VDQQD	Caspase-2	SCCR	*J. Biol. Chem.* **1997**, *272*, 9677-9682
54	Suc-VDVAD	Caspase-2, Caspase-3	SCCR	*J. Biol. Chem.* **1997**, *272*, 9677-9682
55	Suc-DESD	Caspase-2, Caspase-3	SCCR	*J. Biol. Chem.* **1997**, *272*, 9677-9682
56	Suc-DEVD	Caspase-3	RP-MPLC	*J. Biol. Chem.* **1997**, *272*, 9677-9682
57	Suc-GVQVD	Caspase-3, Caspase-6	SCCR	*J. Biol. Chem.* **1997**, *272*, 9677-9682
58	Suc-IETD	Caspase-8, Granzyme B	SCCR	*Cell Death Differ.* **2008**, 15, 322-331
59	Suc-GIETD	Caspase-8, Granzyme B	SCCR	*Cell Death Differ.* **2008**, 15, 322-331
60	Suc-LEHD	Caspase-9	SCCR	*Br. J. Ophthalmol.* **2006**, *90*, 760-764
61	Suc-LLEHD	Caspase-9	SCCR	*Br. J. Ophthalmol.* **2006**, *90*, 760-764
62	Suc-LEED	Caspase-13	SCCR	*Blood* **2004**, *104*, 1998
63	Suc-IEPD	Granzyme B	SCCR	*J. Am. Chem. Soc.* **2020**, *142*, 7075-7082
64	Suc-GIEPD	Granzyme B	SCCR	*J. Am. Chem. Soc.* **2020**, *142*, 7075-7082
65	Suc-EETD		SCCR	
66	Suc-VETD		RP-MPLC	
67	Suc-IKTD		SCCR	
68	Suc-ISTD		SCCR	
69	Suc-IEKD		SCCR	
70	Suc-IEVD		SCCR	
71	Suc-ELEED		SCCR	
72	Suc-EGITD		SCCR	
73	Suc-VWTTD		RP-MPLC	
74	Suc-AAPPD		SCCR	
75	Suc-AAD		SCCR	
76	Suc-LLE	Proteasome	SCCR	*ChemMedChem* **2010**, *5*, 1236-1241
77	Suc-IETE		SCCR	
78	Suc-GDEVE		SCCR	
79	Suc-DYEVE		SCCR	
80	Suc-GIETE		SCCR	
81	Ac-GP	FAP-alpha	SCCR	*bioRxiv* (DOI: 10.1101/2025.08.07.669035)
82	Suc-GPLGP	Amyloid A4 generating enzyme	SCCR	*FEBS Lett.* **1990**, *260*, 131-134
83	Suc-AAA	Elastase	SCCR	*Cell. Rep. Methods* **2024**, *4*, 100688
84	Suc-VIA		SCCR	
85	Suc-AAPAbu	Pancreatic elastase, neutrophil elastase	SCCR	*Cell. Rep. Methods* **2024**, *4*, 100688
86	Suc-AAPV	Elastase	SCCR	*J. Pathol. Microbiol. Immun.* **1992**, *100*, 1073-1080
87	Suc-AAV		SCCR	
88	Suc-AAI		SCCR	
89	Suc-AAH		SCCR	
90	Suc-AAS		SCCR	
91	Suc-EVCit	Cathepsins	RP-MPLC	*Nat. Commun.* **2018**, *9*, 2512
92	Suc-LRGG	SUMO-specific protease	SCCR	*Cell. Rep. Methods* **2024**, *4*, 100688
93	Suc-QTGG	SUMO-specific protease	SCCR	*Cell. Rep. Methods* **2024**, *4*, 100688
94	γGlu	GGT1	SCCR	
95	R	CD13	SCCR	*Cell. Rep. Methods* **2024**, *4*, 100688
96	GP	DPP4	RP-MPLC	*Cell. Rep. Methods* **2024**, *4*, 100688
97	SP	DPP4, DPP9	SCCR	*RSC Chem. Biol.* **2022**, *3*, 859-867
98	HA		SCCR	
99	KA		SCCR	
100	GA		SCCR	
101	YA		SCCR	
102	Ac-A	APEH	SCCR	*J. Am. Chem. Soc.* **2013**, *135*, 6002-6005
103	Ac-M	APEH	SCCR	*J. Am. Chem. Soc.* **2013**, *135*, 6002-6005

※1: Preparation refers to the final process employed to acquire the compounds used in the study. SCCR means that the probes were used after the release from the solid phase without further purification. RP-MPLC means that the probes were purified over reverse-phase MPLC after the release from the solid phase.
※2: References were primarily derived from literature discussing the substrate specificities of 7-amino-4-methylcoumarin (AMC) or *p*-nitrophenylaniline (*p*-NA).

Table S2. Category of proteases and representative enzymes

Category	P1 amino acids	Representative enzymes
Trypsin-like	Arg, Lys	Kallikreins, Cathepsins, Trypsin, Plasmin, Thrombin, Factor Xa, Furin, Urokinase, Enterokinase, Proteasome (trypsin-like), Tryptase, Calpain
Chymotrypsin-like	Phe, Trp, Tyr	Kallikreins, Cathepsins, Chymotrypsin, Chymase, Calpain, Renin, Proteasome (chymotrypsin-like)
Caspase-like	Asp	Caspase-1, Caspases, Granzyme B, Proteasome (caspase-like)
Elastase-like	Ala, Leu, Val	Neutrophil elastase, Pancreatic elastase
Pro endopeptidase	Pro	DPPs, FAPα, Amyloid A4 generating enzyme
SENPs	Gly	SENP1, SENP2

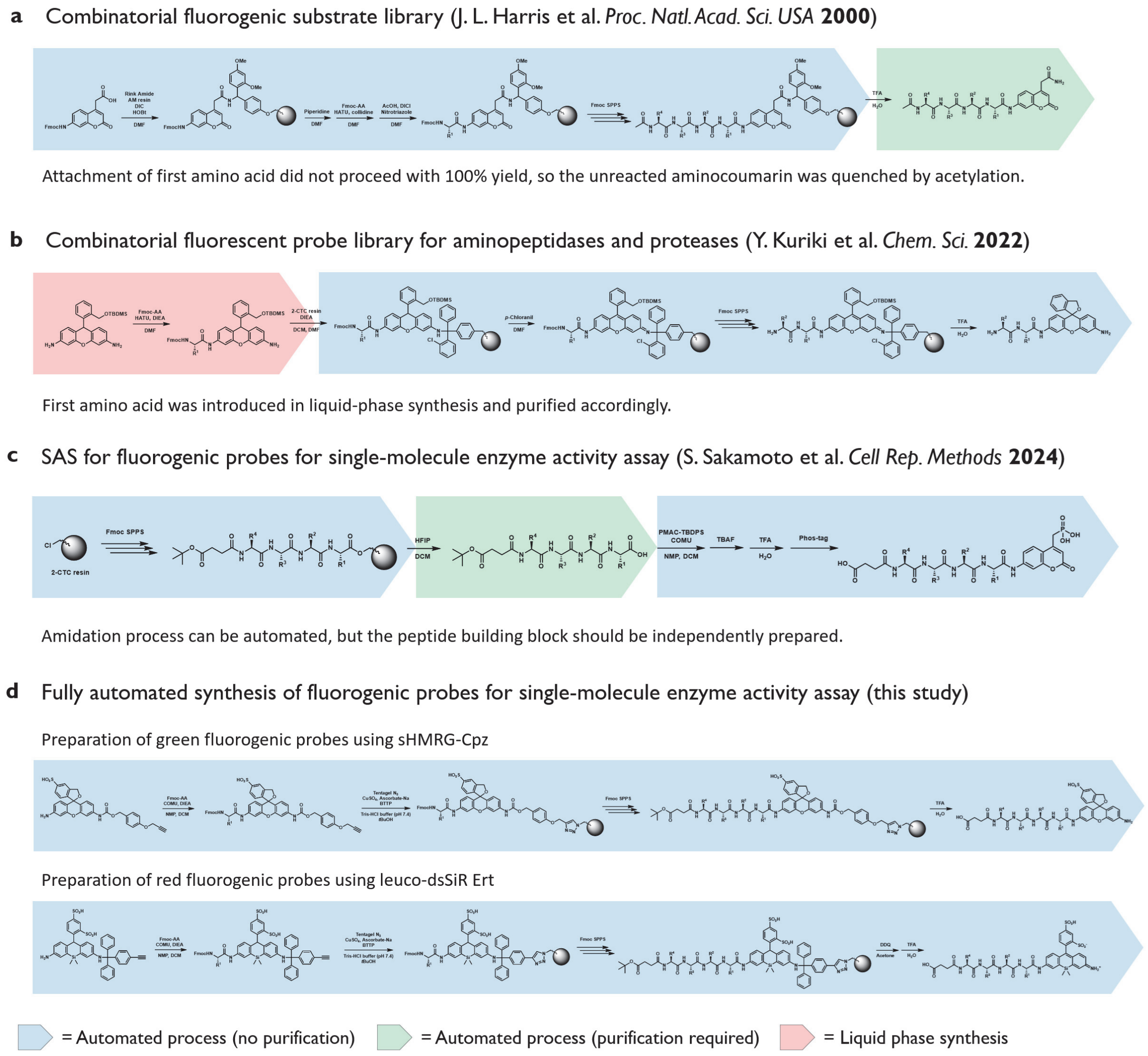

Figure S1. Comparison of methodologies used to prepare the library of fluorogenic substrates for peptidases. (a)-(c) refer to previously reported procedures for the preparation of peptide-modified fluorogenic probes for peptidases[1–3]. (c) represents the SAS strategy, and (d) represents the SCCR strategy employed in this study. Blue highlights indicate steps that can be automated using a peptide synthesizer; yellow highlights indicate steps that can be performed in an automated manner but require manual purification to achieve assay-ready purity (>90% at 500 nm, <1% fluorescent byproducts); and red highlights indicate steps performed using standard organic synthesis procedures.

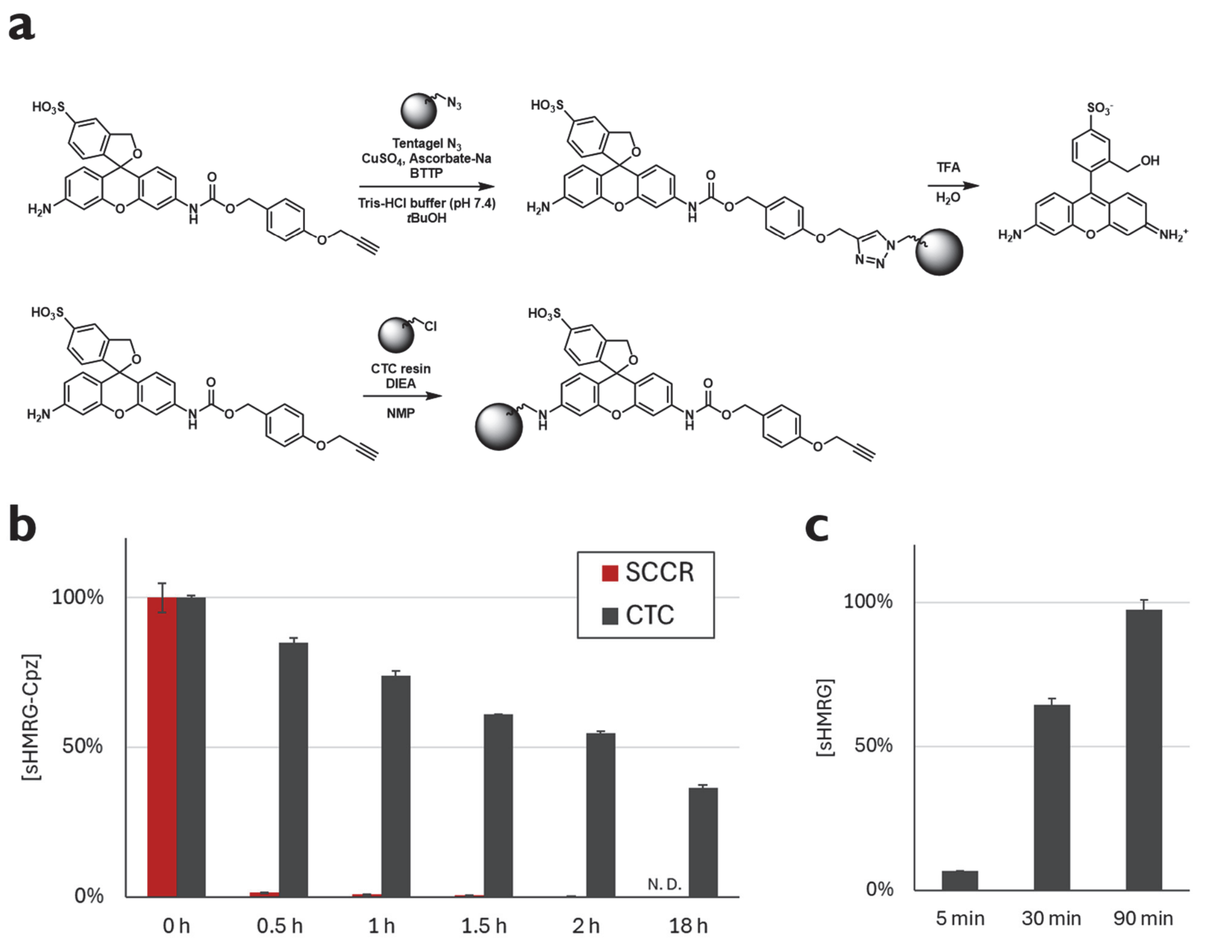

Figure S2. Performance of Cpz/Pzl capturing by azide resin. (a) Structures of sHMRG-Cpz captured by azide resin or CTC resin. (b) Capture of sHMRG-Cpz by azide resin (SCCR; red) and 2-chlorotrityl chloride resin (CTC; gray). For capture by azide resin, sHMRG-Cpz (0.5 μmol) was dissolved in 1 mL of Tris-HCl buffer (100 mM, pH 7.4) containing *t*-BuOH (40%), DMSO (10%), $CuSO_4$ (1 mM), BTTP (3 mM), and sodium ascorbate (3 mM) and incubated with azide resin (0.26 mmol/g, 50 mg) at 25°C for the indicated times. For capture by CTC resin, sHMRG-Cpz (0.5 μmol) was dissolved in 400 μL of NMP and 100 μL of DIEA and incubated with CTC resin (1.55 mmol/g, 30 mg) at 25°C for the indicated times. The sHMRG-Cpz remaining in solution was quantified using LC-MS. Error bars represent standard deviation (S. D.; n = 3). N. D. = not detectable. (c) Release of azide resin–captured sHMRG-Cpz by acidic deprotection. For the beads captured under the conditions described in (c) for 2 h, beads were washed three times with DMF and DCM, and then 90% TFA-10% H_2O (500 μL) was added, and the sample was shaken at 25°C for the indicated times. Released sHMRG was quantified using LC-MS. The recovery rate was calculated by normalization relative to sHMRG-Cpz (0.5 μmol) treated with 90% TFA-10% H_2O (500 μL). Error bars represent S. D. (n = 3).

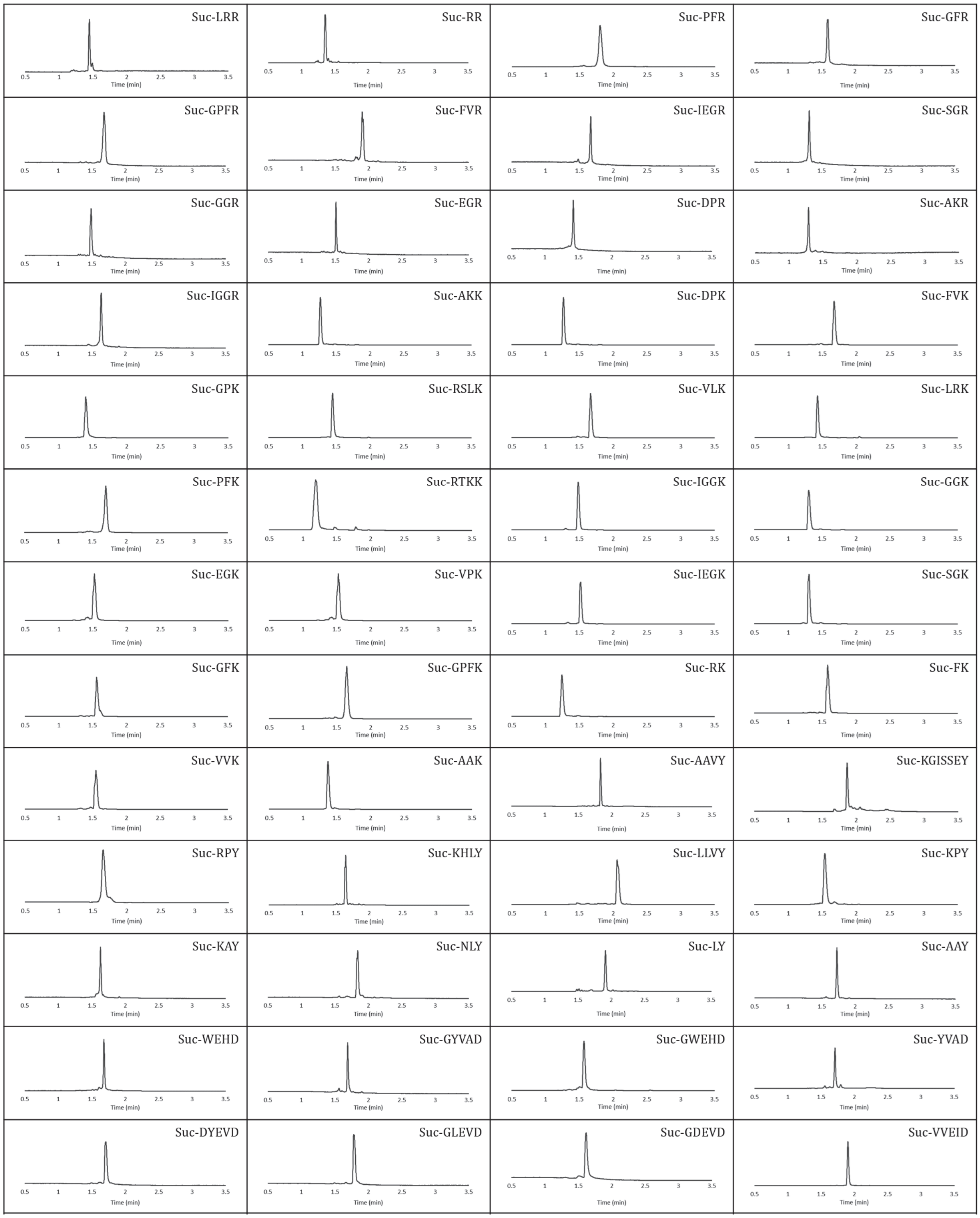

Figure S3. LC chromatograms (absorbance at 500 nm) of sHMRG-based probes #1-#53. Chromatograms for previously reported compounds[4] were not included.

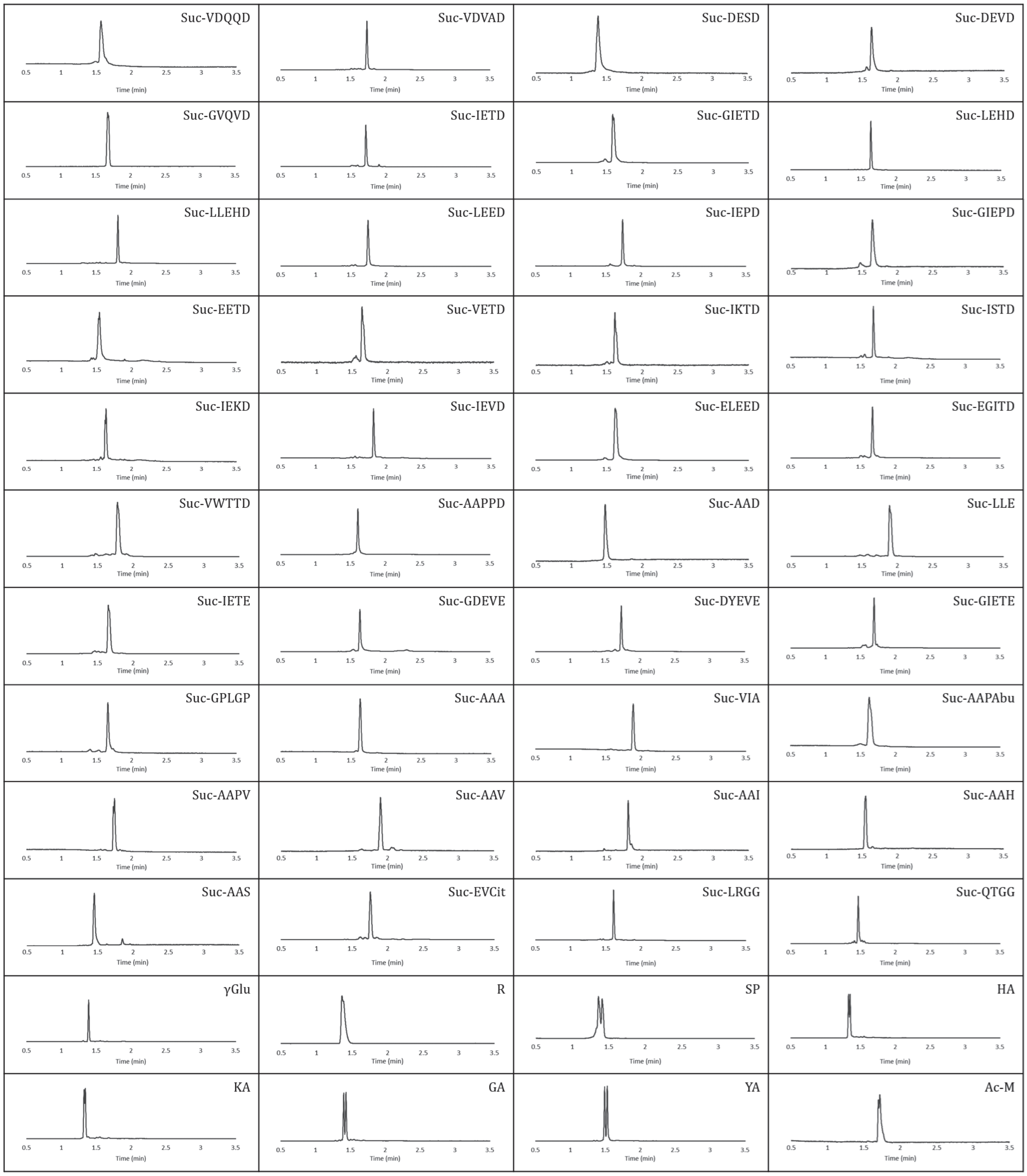

Figure S4. LC chromatograms (absorbance at 500 nm) of sHMRG-based probes #54-#103. Chromatograms for previously reported compounds[2]
were not included.

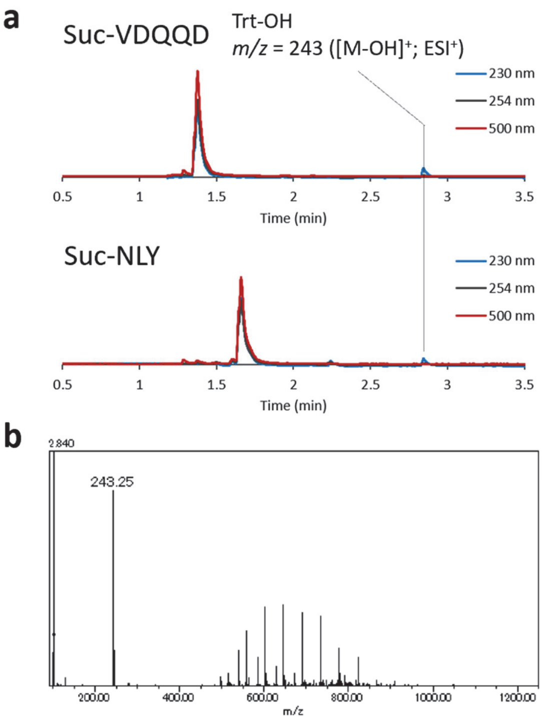

c

Sequence	Yield (%)	Purity (%)	
		500 nm	254 nm
Suc-FVK	63	97	95
Suc-GGK	68	98	95
Suc-AAVY	69	95	92
Suc-NLY	14	93	91
Suc-GYVAD	48	94	93
Suc-VDVAD	53	92	89
Suc-VDQQD	26	98	96
Suc-IETD	57	94	92
Suc-LLE	57	93	88
Suc-AAPAbu	69	97	94
Suc-EVCit	63	92	91
Suc-QTGG	14	91	87

Figure S5. Purity and yield of representative fluorogenic probes. (a) LC chromatograms of representative probes (Suc-VDQQD-sHMRG and Suc-NLY-sHMRG) having amino acids with side-chain protecting groups that generate non-volatile fragments (Trt). The analysis was performed by monitoring at wavelengths of 230 nm, 254 nm, and 500 nm after cleavage from the solid phase. The peak observed at 230 nm corresponds to the Trt-OH fragment. (b) Mass spectrum observed at 2.84 min (m/z = 243.3 $[M\text{-}OH]^+$) of (a). (c) List of yields and purities of 12 representative probes. The yields were calculated from starting material sHMRG-Cpz. The chromatographic purities were determined by peak area integration monitored at 500 nm and 254 nm.

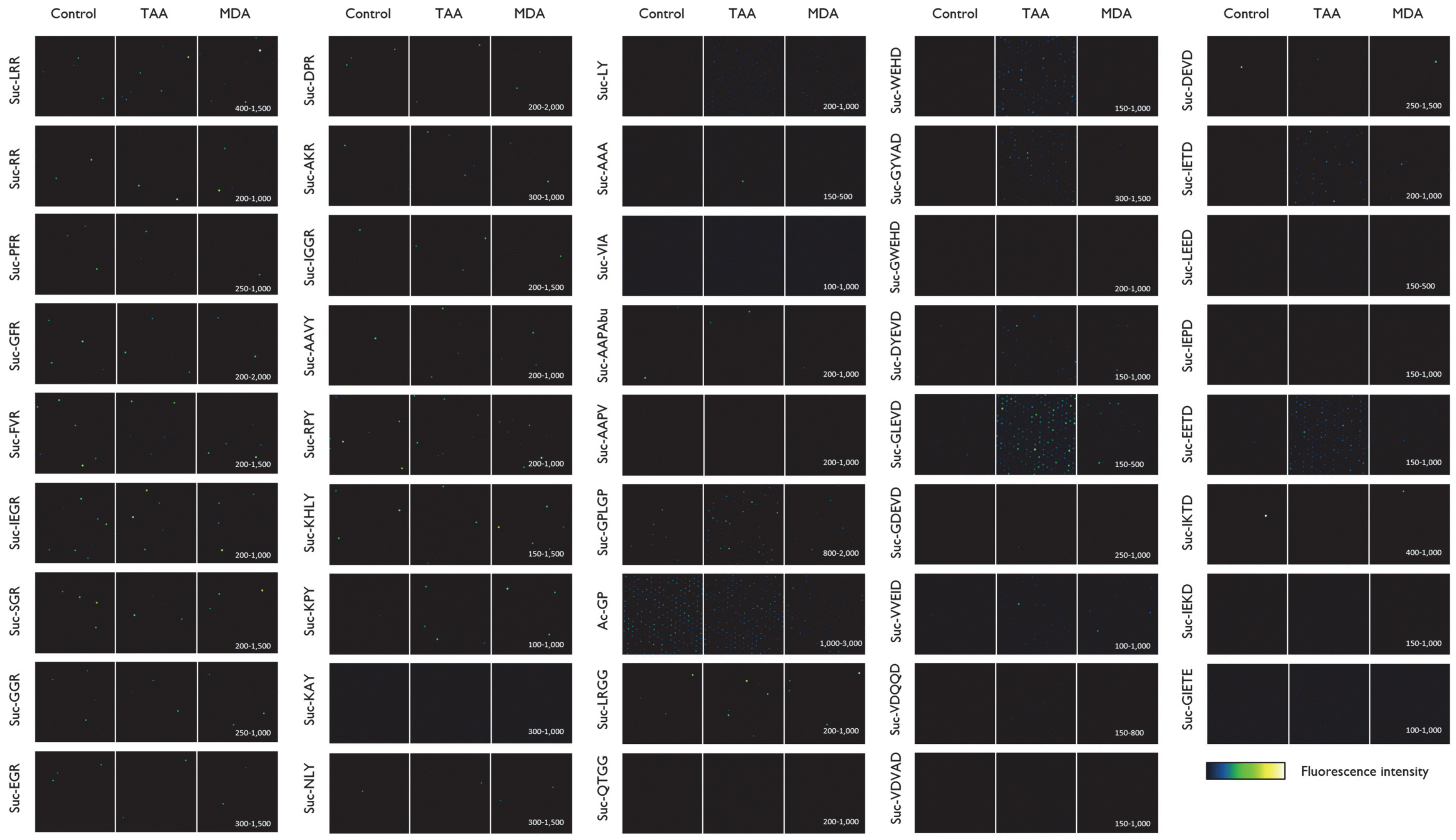

Figure S6. Result of activity-based screening of potential biomarkers of liver damage. The assay was performed by mixing probes (30 μM) with plasma samples (1/100, 1/1,000 for Pro-endopeptidases) in HEPES-Na buffer (100 mM, pH 7.4) containing $CaCl_2$ (1 mM), $MgCl_2$ (1 mM), DTT (1 mM), and Triton X-100 (250 μM) and incubating at 25°C for 18 h.

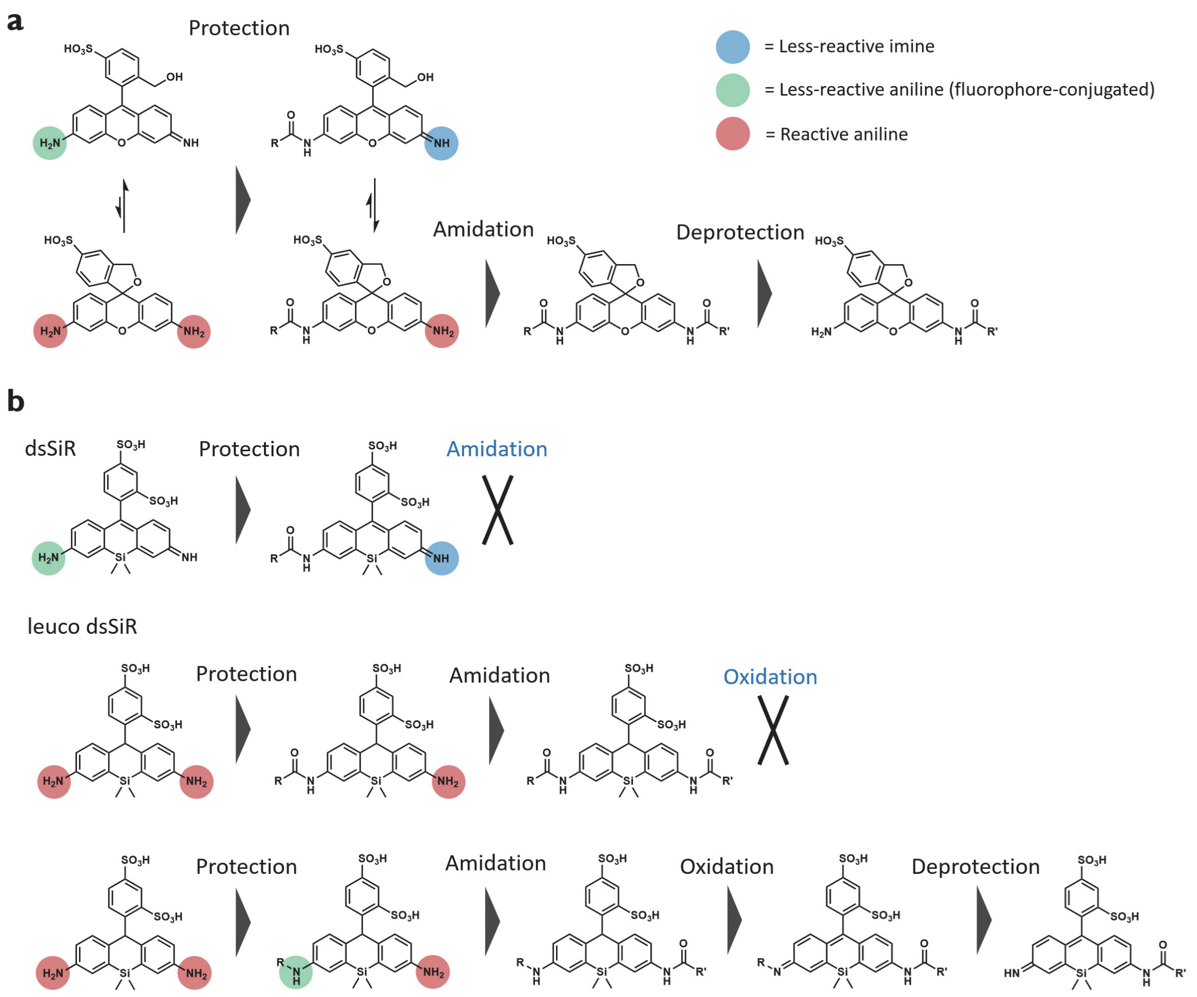

Figure S7. Requirements for appropriate protecting groups to enable reactions at the aniline moieties of rhodamine fluorophores. (a) Protection strategy of sHMRG. Red circles indicate anilines with high reactivity (not part of the extended conjugated system of the fluorophore), yellow circles indicate anilines with low reactivity (within the conjugated system of the fluorophore), and blue circles indicate imines that are unreactive toward amidation. (b) Protection strategy of dsSiR and its leuco form for the design of SCCR-compatible starting materials.

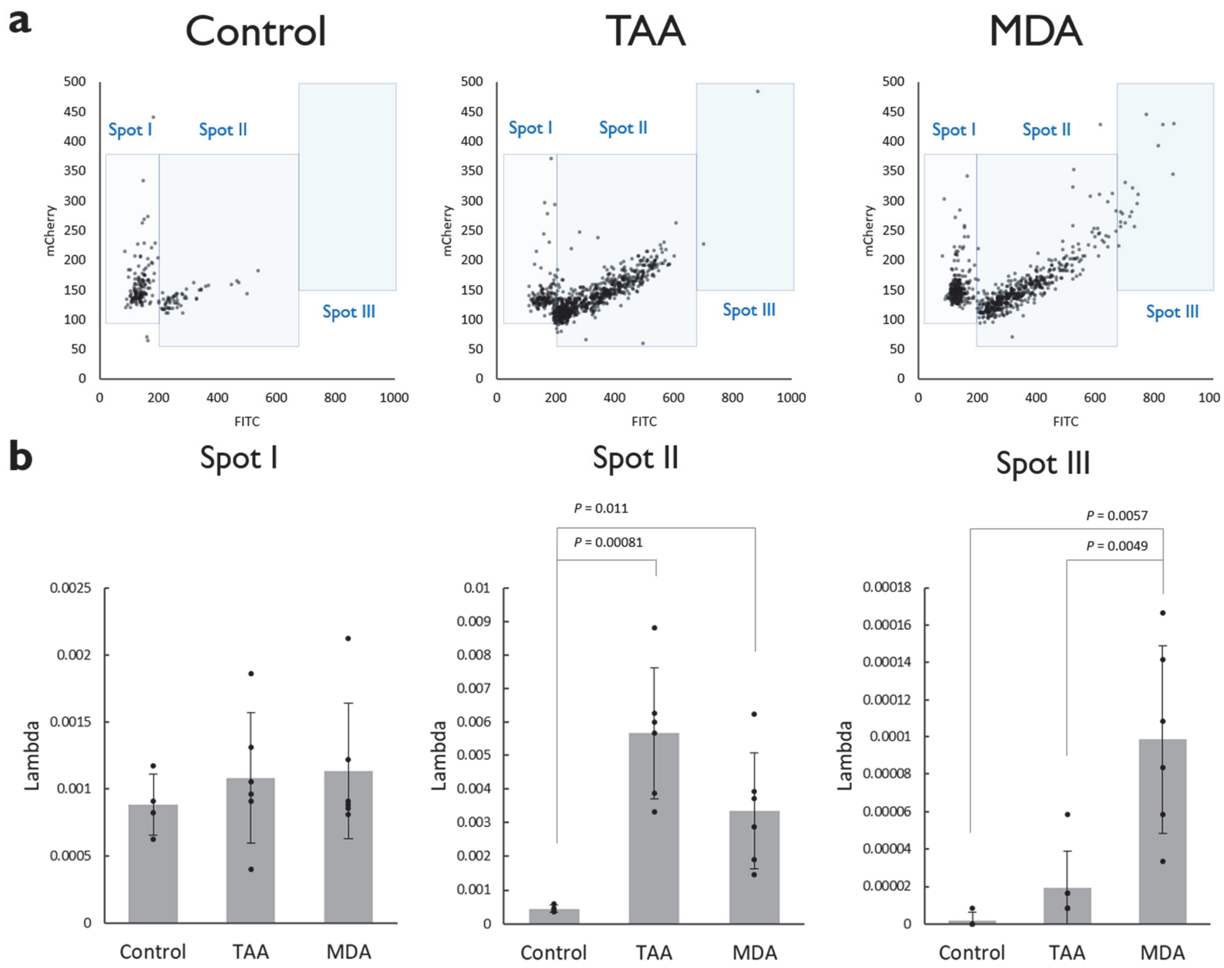

Figure S8. Analysis of activity spots observed for probes targeting kallikrein-like activities. (a) Scattered plot acquired from analyzing the microdevice after loading Suc-KHLY-sHMRG (30 μM), Suc-AAVY-dsSiR (30 μM) and plasma samples (1/100 dilution) in HEPES-Na buffer (100 mM, pH 7.4) containing $CaCl_2$ (1 mM), $MgCl_2$ (1 mM), DTT (1 mM), and Triton X-100 (250 μM) and incubating for 18 h at 25°C. The horizontal and vertical axes correspond to green fluorescence (sHMRG) and red fluorescence (dsSiR), respectively. (b) Quantification of spots observed in the area of spot I-III in (a). n = 4 for control and n = 6 for TAA- or MDA-treated mice. Error bars represent S. D. *P* values were calculated using Student's t-test.

**Name** (Motif)	**Cpz group** (*p*-methoxybenzyl carbamate)	**Pzl group** (PMB group)	**Ert group** (Trt group)
Structures	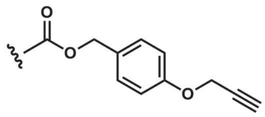	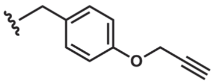	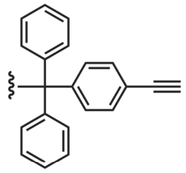
Applicable functional groups	Amines (carbamate)	Amines<br>Alcohol (ether)<br>Carboxylic acid (ester)	Amines<br>Alcohol (ether)<br>Carboxylic acid (ester)
Deprotection condition	Strong acid<br>(e.g., 90% TFA)		Weak acid<br>(e.g., 10% TFA, HFIP)

Figure S9. SCCR-compatible protecting groups reported in this study. The chemical structures, potential target functional groups for protection, and corresponding deprotection conditions are shown for the Cpz, Pzl, and Ert groups.

Supplementary methods for synthesis and characterization of compounds

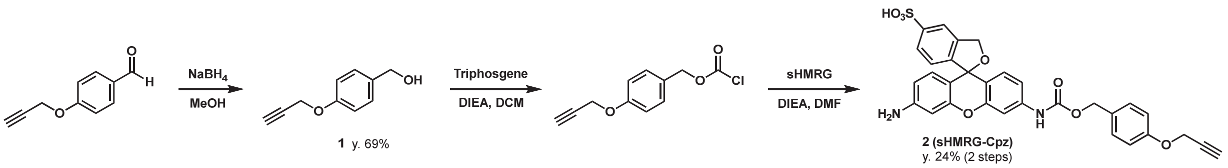

Scheme S1. Preparation of Cpz-OH and protection of sHMRG.

Synthesis of *p*-Propargyloxy benzyl alcohol (Pzl-OH, 1)

p-Propargyloxy benzaldehyde (2.5 g, 15.6 mmol) was dissolved in methanol (10 mL) and stirred on ice. Sodium borohydride ($NaBH_4$, 1.18 g, 31 mmol, 2 eq.) was added portionwise, and the mixture was stirred at 25°C for 30 min. After the reaction, water (20 mL) was added, and the product was extracted three times with dichloromethane (DCM), then washed once with brine. The organic layer was dried over Na_2SO_4 and evaporated to afford a colorless liquid (1.76 g, yield: 69%). The identity of the product was confirmed by comparison of the LC retention time and MS signal with those of an independently synthesized compound via propargylation of *p*-hydroxybenzyl alcohol, as reported.

LRMS (ESI^+): *m/z* = 145 ($[M–OH]^+$)

Synthesis of sHMRG-Cpz (2)

sHMRG is prepared according to the literature. Pzl-OH (150 mg, 0.92 mmol) was dissolved in THF (5 mL), and DIEA (805 µL, 4.62 mmol, 5 equiv.) was added under an Ar atmosphere at 0°C. A solution of triphosgene (69 mg, 0.23 mmol, 0.25 equiv.) in THF (1 mL) was slowly added. The mixture was stirred at 25°C for 30 min to afford a chloroformate solution (A). sHMRG (20 mg, 0.05 mmol) was dissolved in DMSO (1 mL) and DIEA (300 µL), and solution (A) was added in 500 µL portions. The reaction was monitored by HPLC every 10 min, and upon ~50% conversion of sHMRG to the Cpz-protected derivative, the mixture was quenched with water (1 mL) and concentrated under reduced pressure. The residue was dissolved in water and purified by preparative MPLC (eluent: A = 0.1% TFA in H_2O, B = 0.1% TFA in MeCN, gradient from A:B = 95:5 to 5:95 over 15 min). The desired fractions were pooled and lyophilized to give an orange solid (7 mg, yield: 24%).

^{1}H-NMR (400 MHz, CD_3OD): δ 3.45 (s, 1H), 4.33 (s, 2H), 4.71 (s, 2H), 5.18 (s, 2H), 6.7–7.1 (m, 6H), 7.2–7.4 (m, 5H), 7.95 (d, 1H, *J* = 8.2 Hz), 8.16 (s, 1H).

^{13}C NMR (100 MHz, DMSO-d_6): δ55.9, 61.3, 67.2, 78.9, 79.7, 97.7, 104.3, 115.0, 115.4, 117.8, 124.9, 125.7, 128.4, 129.0, 129.2, 130.1, 130.8, 131.1, 133.9, 135.8, 140.8, 150.3, 153.6, 155.6, 157.8, 158.5, 159.7, 162.9.

HRMS (ESI^+): *m/z* Calcd. for $[M+H]^+$, 585.1326, Found, 585.1351 (+2.5 mDa).

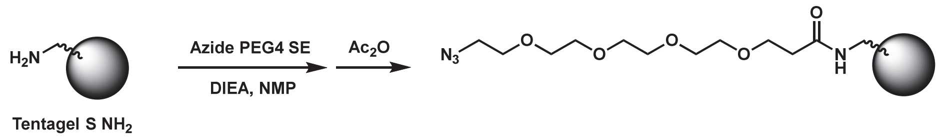

Scheme S2. Preparation of azide resin from Tentagel-NH_2.

Preparation of azide resin from Tentagel-NH_2.

Tentagel S NH_2 (Sigma-Aldrich 86364, 90 µm, 0.26 mmol/g, 600 mg, 156 µmol) was suspended in a mixture of DIEA and NMP (5 mL, 1:2, v/v), followed by the addition of Azide-PEG_4-SE (TCI A2388, 25 mg, 64 µmol). The mixture was stirred at 25°C for 18 h. After the reaction, the beads were washed three times with DMF, then resuspended in a mixture of DIEA and NMP (5 mL, 1:2, v/v), and acetic anhydride (156 µL) was added. The mixture was stirred at 25°C for 3 h. The beads were then washed three times with DMF and three times with DCM, dried under reduced pressure, and stored at 4°C.

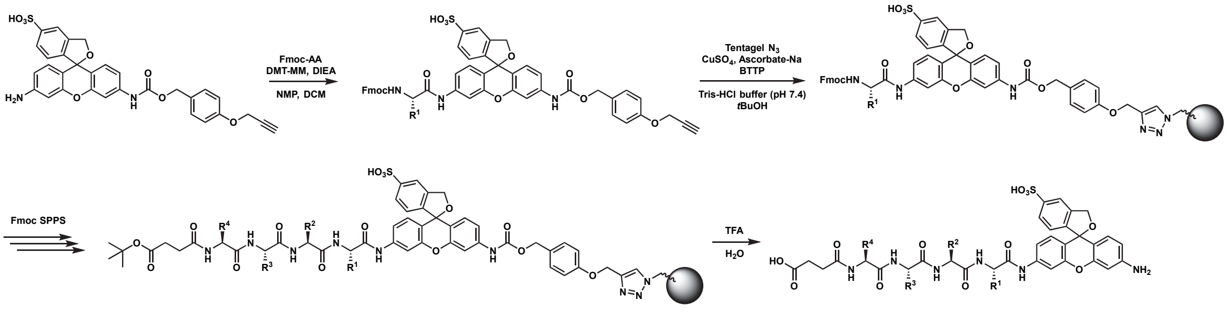

Scheme S3. Synthetic scheme of sHMRG-based probes

Preparation of sHMRG-based probes

All procedures were performed using an automated peptide synthesizer (Syro I, Biotage) equipped with a heating block. The amino acid building blocks (Fmoc-AA) were N^{α}-Fmoc protected, and side chains were protected with standard acid-labile protecting groups (e.g., *t*Bu-, Boc-, Trt-, and Pbf-based protections).

(1) Amidation: sHMRG-Cpz (1 µmol) was mixed with Fmoc-protected amino acid building block (P1 amino acid, 10 µmol), DMT-MM (2.8 mg, 10 µmol) and DIEA (2.8 µL, 15 µmol) in NMP (10 µL) and DCM (500 µL), and the reaction was stirred at 65°C for 2 h.

(2) Capture and wash: Tentagel-azide beads (100 mg) were suspended in Tris-HCl buffer (300 mM, pH 7.4; 300 µL) in the reactor. Reaction mixture of (1) was diluted in *t*-BuOH (400 µL) and added into the reactor. $CuSO_4$ (10 mM in H_2O, 100 µL), TBTA (30 mM in DMSO, 100 µL) and sodium ascorbate (30 mM in H_2O, 100 µL) were added to the reactor and stirred at 25°C for 2 h. The beads were washed six times with DMF.

(3) Peptide elongation: The peptide synthesis was performed by treating beads with conditions for (a) Fmoc deprotection and (b) amino acid coupling, successively until the full peptide sequence was prepared. (a)

Piperidine (40% in DMF; 1200 μL) was added to beads and stirred at 25°C for 3 min. After removing the solvent, piperidine (40% in DMF; 600 μL) and DMF (600 μL) were added and stirred for 12 min. Beads were washed six times with DMF. (b) Fmoc-AA (0.4 M in DMF, 800 μL), HATU (0.4 M in DMF, 840 μL), DIEA (1.6 M in NMP, 400 μL) were added to beads and stirred at 30°C for 40 min. Beads were washed three times with DMF. Mono-*tert*-butyl succinate was used as a building block of Suc capping.

(4) Cleavage: 90% TFA *aq.* (200 μL) was added to the beads and stirred at 25°C for 15 min. The solution was collected, and the beads were washed three times with acetone (200 μL). The combined solution was diluted with H_2O and freeze-dried. For the probes used for validation assay and probes with purity < 90% (500 nm), they were purified over prep. MPLC (C_{18}; H_2O-0.1% TFA/AcCN-0.1% TFA = 95/5 to 0/100 over 15 min). 10 mM DMSO stock solution was prepared by measuring the absorbance of compound in 0.1N HCl aq. and calculating the concentration based on ε of amide-protected HMRG to be 30,000 .

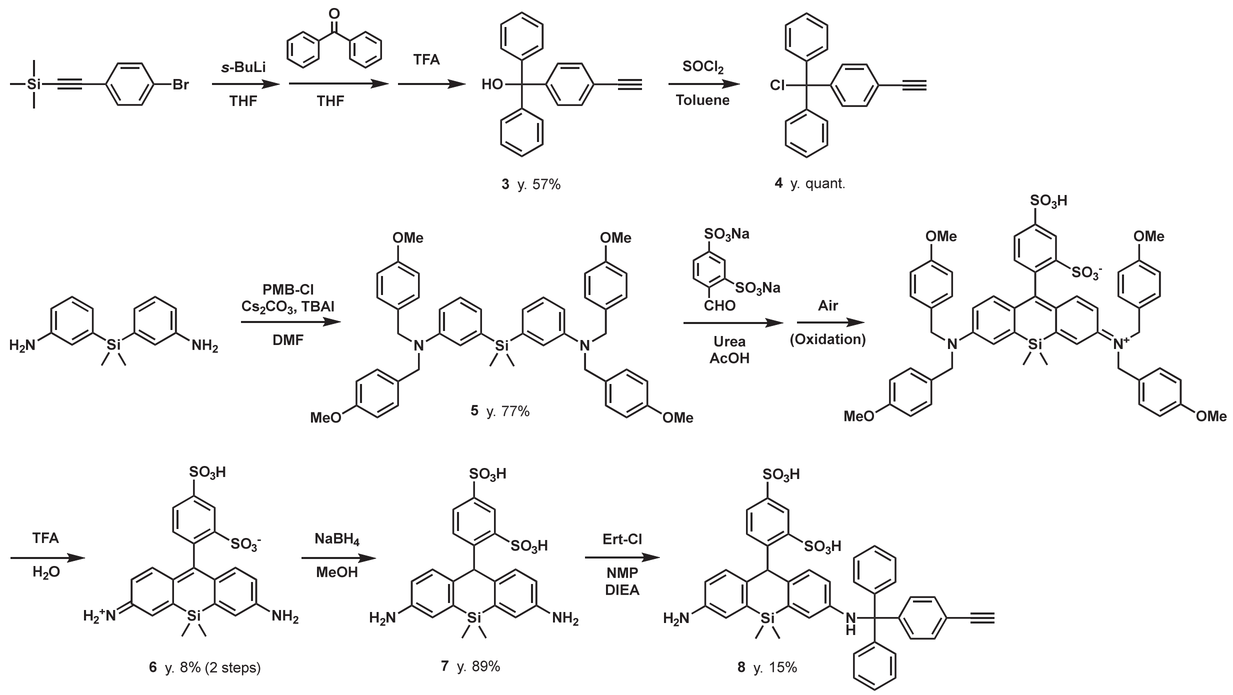

Scheme S4. Preparation of Ert-OH and leuco dsSiR-Ert.

Synthesis of Ethynyl-trityl alcohol (Ert-OH, 3)

(4-Bromophenylethynyl)-trimethylsilane (500 mg, 1.97 mmol) was dissolved in THF (50 mL) and cooled to -78°C under an Ar atmosphere. *sec*-BuLi in hexane (1.3 M, 1.5 mL, 1.97 mmol, 1 eq.) was added dropwise. The reaction mixture was allowed to warm to 25 °C and stirred for 30 min, then cooled again to -78 °C. A solution of benzophenone (360 mg, 1.97 mmol, 1 eq.) in THF (2 mL) was added dropwise, and the mixture was stirred at 25 °C for 2 h. Water was then added, and the product was extracted three times with DCM and washed once with brine. The organic layer was dried over Na_2SO_4 and concentrated under reduced pressure. The crude product was purified by silica gel column chromatography (eluent: hexane–DCM, 100:0 to 0:100 over 15 min)

to give **3** as a colorless liquid (320 mg, yield: 57%).

^{1}H-NMR (400 MHz, $CDCl_3$): δ 2.77 (s, 1H), 3.05 (s, 1H), 7.3 (m, 12H), 7.45 (d, 2H, *J* = 8.2 Hz)

Synthesis of Ethynyl-trityl chloride (Ert-Cl, 4)

Ert-OH (50 mg, 0.18 mmol) was dissolved in toluene (1 mL), and thionyl chloride ($SOCl_2$, 76 μL, 1.1 mmol, 6 eq.) was added. The reaction was refluxed for 18 h. After cooling, excess toluene was added and solvents were removed *in vacuo*. The crude product was used directly in the next step.

^{1}H-NMR (400 MHz, $CDCl_3$): δ 3.08 (s, 1H), 7.2 (m, 6H), 7.3 (m, 6H), 7.42 (d, 2H, *J* = 8.2 Hz)

Preparation of 5

3,3'-(Dimethylsilanediyl)dianiline was prepared according to the literature . 3,3'-(Dimethylsilanediyl)dianiline (400 mg, 1.65 mmol), *p*-methoxybenzyl chloride (1.1 mL, 8.25 mmol, 5 eq.), DIEA (1.4 mL, 8.25 mmol, 5 eq.), tetrabutylammonium iodide (30 mg, 0.08 mmol, 0.05 eq.) were dissolved in DMF (2 mL) and stirred at 70°C for 6 h. The reaction was diluted by DCM and directly subjected to column for silica gel column chromatography (eluent: hexane/AcOEt) to afford compound **5** (914 mg, yield: 77%).

^{1}H-NMR (400 MHz, $CDCl_3$): δ 0.32 (s, 6H), 3.77 (s, 12H), 4.49 (s, 8H), 6.72 (dd, 2H, *J* = 8.6, 2.4 Hz), 6.76 (d, 2H, *J* = 8.4 Hz), 6.81 (d, 8H, *J* = 8.4 Hz), 6.91 (d, 2H, *J* = 2.4 Hz), 7.1-7.2 (m, 10H).

^{13}C-NMR (100 MHz, $CDCl_3$): δ -2.43, 53.7, 55.4, 113.8, 113.0, 114.0, 118.4, 124.4, 128.1, 128.6, 130.8, 139.0, 148.5, 158.6.

LRMS (ESI^+): *m/z* = 723 ($[M + H]^+$)

Synthesis of dsSiR (6)

Compound **5** (490 mg, 0.68 mmol), 4-formylbenzene-1,3-disulfonic acid disodium salt (142 mg, 0.81 mmol, 1.2 eq.) and urea (30 mg, 0.51 mmol, 0.75 eq.) were dissolved in acetic acid (10 mL) and stirred at 95°C for 20 h in open air. LC-MS-based reaction monitoring suggested complete consumption of the starting material, formation of the oxidated fluorophore, and partial deprotection of PMB groups. The solvents were removed *in vacuo*, and the products were purified over silica gel column chromatography (DCM/MeOH 100/0 to 20/80 over 20 min). Fractions containing tetra-PMB and tri-PMB dsSiR were collected and the solvent was removed *in vacuo*. To the remaining TFA (2 mL) was added to the residue, and the mixture waas stirred at 25°C for 40 min. LC-MS-based reaction monitoring suggested formation of dsSiR and its mono-PMB form. Solvent was removed *in vacuo*, and the products were purified over prep. MPLC (eluent: A = 0.1% triethylamine in H_2O, B = MeOH, gradient from A:B = 95:5 to 5:95 over 15 min) to afford dsSiR (25 mg, yield: 8%).

^{1}H NMR (400 MHz, CD_3OD): δ 0.47 (s, 6H), 6.54 (d, 2H, *J* = 8.4 Hz), 6.93 (d, 2H, *J* = 8.4 Hz), 7.16 (s, 2H), 7.20 (d, 1H, *J* = 7.2 Hz), 7.96 (d, 1H, *J* = 7.2 Hz), 8.44 (s, 1H).

HRMS (ESI^+): *m/z* Calcd. for $[M+H]^+$, 489.0605, Found, 489.0647 (+4.2 mDa).

Synthesis of leuco-dsSiR (7)

dsSiR (48 mg, 0.10 mmol) was dissolved in methanol (3 mL) and sodium borohydride (22 mg, 0.59 mmol)

was added, and the reaction was stirred at 25°C for 2 h. H_2O was added, and the solvent was removed under reduced pressure. The crude product was purified over preparative MPLC (eluent: A = 0.1% triethylamine in H_2O, B = MeOH, gradient from A:B = 95:5 to 5:95 over 15 min), and the combined fractions were freeze-dried to afford leuco dsSiR (43 mg, yield: 89%).
^{1}H NMR (400 MHz, CD_3OD/D_2O = 1:1): δ 0.52 (s, 3H), 0.53 (s, 3H), 6.59 (dd, *J* = 8.4, 2.4 Hz, 2H), 6.98 (d, *J* = 8.4 Hz, 2H), 7.21 (d, *J* = 2.4 Hz, 2H), 7.26 (d, *J* = 7.6 Hz, 1H), 8.01 (dd, *J* = 7.6, 2.0 Hz, 1H), 8.48 (d, *J* = 2.0 Hz, 1H).
HRMS (ESI$^+$): *m/z* = 491 ($[M + H]^+$)

Synthesis of leuco dsSiR-Ert (8)

Leuco dsSiR (20 mg, 0.04 mmol) was dissolved in NMP (20 µL). A solution of Ert-Cl (6.2 mg, 0.02 mmol, 0.5 eq.) and DIEA (36 µL, 0.2 mmol, 5 eq.) in DCM (100 µL) was added dropwise. DCM was removed under reduced pressure over 30 min. The resulting residue was dissolved in water and purified by preparative MPLC (eluent: A = 0.1% triethylamine in H_2O, B = MeOH, gradient from A:B = 95:5 to 5:95 over 15 min). Fractions containing the desired product were pooled. Triethylamine (50 µL) was added to the solution and lyophilized to afford leuco dsSiR Ert (4.5 mg, yield: 15%).
^{1}H NMR (400 MHz, DMSO-d_6): δ 0.52 (s, 6H), 3.06 (s, 1H), 4.09 (s, 1H), 6.32 (dd, 1H, *J* = 8.0, 2.4 Hz), 6.37 (d, 1H, *J* = 2.4 Hz), 6.48 (dd, 1H, *J* = 8.0, 2.4 Hz), 6.49 (d, 2H, *J* = 8.4 Hz), 6.53 (s, 1H), 6.58 (d, 1H, *J* = 2.4 Hz), 7.1-7.2 (m, 3H), 7.2-7.3 (m, 8H), 7.3-7.4 (m, 4H), 8.08 (d, 1H, *J* = 2.0 Hz).
^{13}C NMR (100 MHz, DMSO-d_6): δ 1.0, 46.3, 70.9, 81.2, 83.8, 116.6, 117.5, 119.0, 120.1, 120.2, 125.3, 126.2, 127.0, 128.3, 129.4, 129.7, 130.8, 130.9, 131.1, 131.6, 132.2, 132.3, 138.1, 138.7, 143.8, 143.9, 144.0, 145.6, 145.7, 146.9, 148.1.
LRMS (ESI$^-$): *m/z* = 755 ($[M–H]^+$)

Preparation of dsSiR-based probes

All procedures were performed using an automated peptide synthesizer (Syro I, Biotage) equipped with a heating block. The synthetic scheme is shown in **Figure 4a**.

(1) Amidation: leuco-dsSiR Ert (1 µmol) was mixed with Fmoc-protected amino acid building block (P1 amino acid, 10 µmol), COMU (4.3 mg, 10 µmol) and DIEA (5.6 µL, 30 µmol) in NMP (10 µL) and DCM (500 µL), and the reaction was stirred at 65°C for 2 h.
(2) Capture and wash: Tentagel-azide beads (100 mg) were suspended in Tris-HCl buffer (300 mM, pH 7.4; 300 µL) in the reactor. Reaction mixture of (1) was diluted in *t*-BuOH (400 µL) and added into the reactor. $CuSO_4$ (10 mM in H_2O, 100 µL), TBTA (30 mM in DMSO, 100 µL) and sodium ascorbate (30 mM in H_2O, 100 µL) were added to the reactor and stirred at 25°C for 2 h. The beads were washed six times with DMF.
(3) Peptide elongation: The peptide synthesis was performed by treating beads with conditions for (a) Fmoc deprotection and (b) amino acid coupling, successively until the full peptide sequence was prepared. (a) Piperidine (40% in DMF; 1200 µL) was added to beads and stirred at 25°C for 3 min. After removing the solvent, piperidine (40% in DMF; 600 µL) and DMF (600 µL) were added and stirred for 12 min. Beads

were washed six times with DMF. (b) Fmoc-AA (0.4 M in DMF, 800 μL), HATU (0.4 M in DMF, 840 μL), DIEA (1.6 M in NMP, 400 μL) were added to beads and stirred at 30°C for 40 min. Beads were washed three times with DMF. Mono-*tert*-butyl succinate was used as a building block of Suc capping.

(4) Oxidation: 2,3-Dichloro-5,6-dicyano-1,4-benzoquinone (DDQ) (10 mg, 0.044 mmol) dissolved in acetone (1 mL) was added to the beads and stirred at 25°C for 3 h. Beads were washed six times with acetone.

(5) Cleavage: 90% TFA *aq.* (200 μL) was added to the beads and stirred at 25°C for 15 min. The solution was collected, and the beads were washed three times with acetone (200 μL). The combined solution was diluted with H_2O and freeze-dried. For the probes used for validation assay and probes with purity < 90% (500 nm and 600 nm), they were purified over prep. MPLC (C_{18}; H_2O-0.1% TFA/AcCN-0.1% TFA = 95/5 to 0/100 over 15 min).

Suc-AAVY-dsSiR

LC Chromatogram was monitored at 500 nm (H_2O-0.1% TFA/AcCN-0.1% TFA = 95/5 to 0/100, 3.5 min).

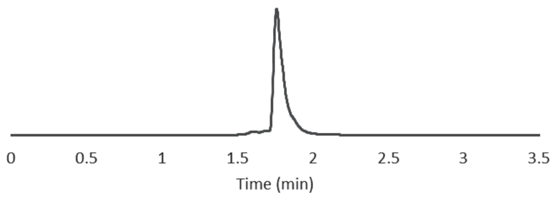

Settings of automated peptide synthesizer and preparation of reagents

The settings were configured for the Syro I automated peptide synthesizer (Biotage) operated via the Syro software (version 2.0.295). Reaction Vial 1 (RV1), dedicated to the solid-phase capture and reaction, was equipped with a standard 10 mL reactor (MultiSynTech GmbH V100TF073). Reaction Vial 2 (RV2), dedicated to the liquid-phase reaction, was equipped with a 5 mL plastic tube (Eppendorf 0030119401). In the Robot Setup, the Z-cone and Z-max values for RV2 were set to 1900. This ensures that the liquid-phase intermediates can be reliably aspirated from the bottom of the tube.

Reactions for preparation of sHMRG-based probes

Reaction
- Reaction 2 h; 65°C; Vortex 1 min; Break 1 min

Beads capture
- Fill [a] 300 μL -> RV1
- Fill [b] 200 μL -> RV2
- Reaction 30 s; 25°C; Vortex 1 min; Break 1 min
- Fill RV2 200 μL -> RV1
- Fill [b] 200 μL -> RV2
- Reaction 30 s; 25°C; Vortex 1 min; Break 1 min
- Fill RV2 200 μL -> RV1
- Fill [c] 100 μL -> RV1
- Reaction 10 s; 25°C; Vortex 1 min; Break 1 min
- Fill [d] 100 μL -> RV1
- Reaction 10 s; 25°C; Vortex 1 min; Break 1 min
- Fill [e] 100 μL -> RV1
- Reaction 1 h; 25°C; Vortex 10 s; Break 1 min
- Fill [f] 100 μL -> RV1
- Reaction 30 min; 25°C; Vortex 10 s; Break 1 min
- Empty 1 min
- Wash 6 cycles
  - Fill [DMF] 1000 μL -> RV1
  - Reaction 30 s; 25°C; Vortex 10 s; Break 1 min
  - Empty 30 s
- Fmoc deprotection
  - Fill [Piperidine/DMF] 1200 μL -> RV1
  - Reaction 3 min; 25°C; Vortex 10 s; Break 1 min
  - Empty 30 s
  - Fill [Piperidine/DMF] 600 μL -> RV1
  - Fill [DMF] 600 μL -> RV1
  - Reaction 12 min; 25°C; Vortex 10 s; Break 1min
  - Empty 10 s

Amino acid elongation n cycles
- Condensation
  - Fill [AA] 800 μL -> RV1
  - Fill [HATU/DMF] 840 μL -> RV
  - Fill [DIPEA/NMP] 400 μL -> RV
  - Reaction 40 min; 25°C; Vortex 15 s; Break 2 min
  - Empty 30 s

Fmoc deprotection

Fill [Piperidine/DMF] 1200 µL -> RV1
Reaction 3 min; 25°C; Vortex 10 s; Break 1 min
Empty 30 s
Fill [Piperidine/DMF] 600 µL -> RV1
Fill [DMF] 600 µL -> RV1
Reaction 12 min; 25°C; Vortex 10 s; Break 1 min
Empty 10 s

Reagents for preparation of sHMRG-based probes

RV1		
	Tentagel-azide	100 mg
RV2		
	sHMRG-Cpz	5 µmol
	Fmoc-AA	10 µmol
	DMT-MM	10 µmol
	DIEA	2.8 µL
	NMP	10 µL
	DCM	500 µL
Amino acids		
	[AA]	0.4 M
System liquids		
	[Piperidine/DMF]	40% (v/v)
	[DMT-MM/MeOH]	0.4 M
	[DIPEA/NMP]	1.6 M
Reagents		
[a]	Tris-HCl buffer (pH 7.4)	300 mM
[b]	*t*-BuOH	
[c]	$CuSO_4/H_2O$	10 mM
[d]	TBTA/DMSO	30 mM
[e]	Sodium ascorbate/H_2O	30 mM

Supplementary references

(1) Harris, J. L.; Backes, B. J.; Leonetti, F.; Mahrus, S.; Ellman, J. A.; Craik, C. S. Rapid and General Profiling of Protease Specificity by Using Combinatorial Fluorogenic Substrate Libraries. *Proc. Natl. Acad. Sci. USA* **2000**, *97* (14), 7754–7759.

(2) Kuriki, Y.; Yoshioka, T.; Kamiya, M.; Komatsu, T.; Takamaru, H.; Fujita, K.; Iwaki, H.; Nanjo, A.; Akagi, Y.; Takeshita, K.; Hino, H.; Hino, R.; Kojima, R.; Ueno, T.; Hanaoka, K.; Abe, S.; Saito, Y.; Nakajima, J.; Urano, Y. Development of a Fluorescent Probe Library Enabling Efficient Screening of Tumour-Imaging Probes Based on Discovery of Biomarker Enzymatic Activities. *Chem. Sci.* **2022**, *13*, 4474–4481. https://doi.org/10.1039/D1SC06889J.

(3) Sakamoto, S.; Hiraide, H.; Minoda, M.; Iwakura, N.; Suzuki, M.; Ando, J.; Takahashi, C.; Takahashi, I.; Murai, K.; Kagami, Y.; Mizuno, T.; Koike, T.; Nara, S.; Morizane, C.; Hijioka, S.; Kashiro, A.; Honda, K.; Watanabe, R.; Urano, Y.; Komatsu, T. Identification of Activity-Based Biomarkers for Early-Stage Pancreatic Tumors in Blood Using Single-Molecule Enzyme Activity Screening. *Cell Rep. Methods* **2024**, *4*, 100688. https://doi.org/10.1016/j.crmeth.2023.100688.

(4) Sakamoto, S.; Hiraide, H.; Mizuno, T.; Minoda, M.; Nagano, N.; Suzuki, M.; Iwakura, N.; Kashiro, A.; Nara, S.; Morizane, C.; Hijioka, S.; Honda, K.; Kagami, Y.; Watanabe, R.; Urano, Y.; Komatsu, T. Development of Single-Molecule Protease Activity Analysis Platform to Elucidate Disease-Related Alterations of Circulating Proteoform Signatures. *Cell Biomater.* **2026**, *2*, 100456. https://doi.org/10.1101/2025.08.07.669035.

(5) Ge, C.; Zhu, J.; Ye, H.; Wei, Y.; Lei, Y.; Zhou, R.; Song, Z.; Yin, L. Rational Construction of Protein-Mimetic Nano-Switch Systems Based on Secondary Structure Transitions of Synthetic Polypeptides. *J. Am. Chem. Soc.* **2023**, *145* (20), 11206–11214. https://doi.org/10.1021/jacs.3c01156.

(6) Minoda, M.; Mizuno, T.; Iwasaka, T.; Kusuhara, H.; Kagami, Y.; Sakamoto, S.; Nagano, N.; Hori, C.; Honda, K.; Urano, Y.; Komatsu, T. Synthesis Based on Covalent Capture and Release (SCCR): A Programmable Strategy for Automated Preparation of Protease-Activatable Molecules. *bioRxiv* **2026**, 2026.02.07.704608. https://doi.org/10.64898/2026.02.07.704608.

(7) Sakabe, M.; Asanuma, D.; Kamiya, M.; Iwatate, R. J.; Hanaoka, K.; Terai, T.; Nagano, T.; Urano, Y. Rational Design of Highly Sensitive Fluorescence Probes for Protease and Glycosidase Based on Precisely Controlled Spirocyclization. *J. Am. Chem. Soc.* **2013**, *135* (1), 409–414. https://doi.org/10.1021/ja309688m.

(8) Grimm, J. B.; Tkachuk, A. N.; Patel, R.; Hennigan, S. T.; Gutu, A.; Dong, P.; Gandin, V.; Osowski, A. M.; Holland, K. L.; Liu, Z. J.; Brown, T. A.; Lavis, L. D. Optimized Red-Absorbing Dyes for Imaging and Sensing. *J. Am. Chem. Soc.* **2023**, *145* (42), 23000–23013. https://doi.org/10.1021/jacs.3c05273.
